# Differential Metabolic Rearrangements after Cold Storage Are Correlated with Chilling Injury Resistance of Peach Fruits

**DOI:** 10.3389/fpls.2016.01478

**Published:** 2016-09-30

**Authors:** Claudia A. Bustamante, Laura L. Monti, Julieta Gabilondo, Federico Scossa, Gabriel Valentini, Claudio O. Budde, María V. Lara, Alisdair R. Fernie, María F. Drincovich

**Affiliations:** ^1^Centro de Estudios Fotosintéticos y Bioquímicos, Facultad de Ciencias Bioquímicas y Farmacéuticas, Universidad Nacional de RosarioRosario, Argentina; ^2^Estación Experimental San Pedro, Instituto Nacional de Tecnología AgropecuariaSan Pedro, Argentina; ^3^Max-Planck-Institut für Molekulare PflanzenphysiologiePotsdam-Golm, Germany; ^4^Consiglio per la Ricerca in Agricoltura e l'Analisi dell'Economia Agraria, Centro di Ricerca per la FrutticolturaRome, Italy

**Keywords:** chilling injury, cold, metabolome, peach fruit, *Prunus persica*, varieties

## Abstract

Reconfiguration of the metabolome is a key component involved in the acclimation to cold in plants; however, few studies have been devoted to the analysis of the overall metabolite changes after cold storage of fruits prior to consumption. Here, metabolite profiling of six peach varieties with differential susceptibility to develop mealiness, a chilling-injury (CI) symptom, was performed. According to metabolic content at harvest; after cold treatment; and after ripening, either following cold treatment or not; peach fruits clustered in distinct groups, depending on harvest-time, cold treatment, and ripening state. Both common and distinct metabolic responses among the six varieties were found; common changes including dramatic galactinol and raffinose rise; GABA, Asp, and Phe increase; and 2-oxo-glutarate and succinate decrease. Raffinose content after long cold treatment quantitatively correlated to the degree of mealiness resistance of the different peach varieties; and thus, raffinose emerges as a candidate biomarker of this CI disorder. Xylose increase after cold treatment was found only in the susceptible genotypes, indicating a particular cell wall reconfiguration of these varieties while being cold-stored. Overall, results indicate that peach fruit differential metabolic rearrangements due to cold treatment, rather than differential metabolic priming before cold, are better related with CI resistance. The plasticity of peach fruit metabolism renders it possible to induce a diverse metabolite array after cold, which is successful, in some genotypes, to avoid CI.

## Introduction

When plants are exposed to cold, a highly complex response program switches on, which results in a global reconfiguration of both the transcriptome and metabolome. Cold-responsive genes encode a diverse array of proteins with putative roles in cold tolerance, which allow survival in the case of cold-tolerant plants. Given that low temperature is one of the major abiotic stresses, which limits both crop productivity and geographical distribution, the molecular basis of cold tolerance and acclimation has been extensively studied using a range of different approaches (Thomashow, 1999, 2001, 2010; Stitt and Hurry, 2002; Xiong et al., 2002; Cook et al., 2004; Kaplan et al., 2004, 2007; Hannah et al., 2005; Lee et al., 2005; Zhu et al., 2007; Guy et al., 2008; Usadel et al., 2008; Heidarvand and Amiri, 2010; Tarkowski and Van den Ende, 2015). These studies facilitated the identification of key players, including several transcription factors, involved in the response and acclimation to cold. However, due to the intricacy of the plant response to low temperatures, several questions remain still open when trying to design plants with increased freezing tolerance (Thomashow, 2010; Knight and Knight, 2012; Megha et al., 2014).

Despite the damage that low temperature can produce in plants in the case of an unsuccessful acclimation, the application of cold after the harvest of many commodities is a widespread technology used to delay decay and slow ripening. In the case of fleshy fruits, cold storage is effective in extending shelf-life, and preserving fruit quality properties; however, it can lead to the development of a disorder, known as chilling injury (CI; Lyons, 1973; Saltveit, 2000). CI is characterized by different symptoms depending on the fruit and the time of storage. In the case of peach fruits, CI symptoms mainly develop during fruit ripening after cold storage, so this problem is not perceived until the fruit reaches consumers (Lurie and Crisosto, 2005; Pedreschi and Lurie, 2015). Hence, the molecular reconfiguration that takes place during cold storage impacts on the way fruits ripen during the following shelf-life, situation that greatly limits commercialization of these fruits. One of the principal phenotypic expressions of CI in peach is flesh mealiness, which is the consequence of altered cell wall metabolism resulting in a gel-like texture (Brummell et al., 2004; Fruk et al., 2014).

In view of the relevance of the disorders originated by cold when peach fruits are stored, several different approaches have been used in order to identify genes associated to cold response and/or involved in mealiness development. Transcriptomic studies after cold storage using single genotypes (González-Agüero et al., 2008; Ogundiwin et al., 2008; Vizoso et al., 2009; Pavez et al., 2013) or contrasting genotypes with differential susceptibility to cold (Falara et al., 2011; Dagar et al., 2013; Pons et al., 2014, 2015) have been powerful approaches in order to dissect the molecular mechanisms involved in peach cold tolerance. In addition, proteomic studies have also identified key proteins involved in the protection to cold or in the development of CI (Lara et al., 2009; Dagar et al., 2010; Nilo et al., 2010; Zhang et al., 2010; Almeida et al., 2016); while genomic studies have allowed the mapping of a number of quantitative trait loci (QTL) for CI in the peach genome (Ogundiwin et al., 2009; Cantín et al., 2010; Dhanapal et al., 2012; Martinez-Garcia et al., 2012; Nuñez-Lillo et al., 2015). These studies have indicated that CI is a multigenic quantitative trait, and thus, genetic engineering of CI tolerance is a very difficult challenge.

In contrast to transcriptomic data, there are fewer reports on untargeted metabolomic studies in peach fruit (Lara and Drincovich, 2012; Shiratake and Suzuki, 2016). However, considering that cold causes dramatic changes in plant metabolic content, and because fruits are part of human diet, the information about what happens in the fruit metabolome after cold storage is crucial. Moreover, changes in metabolites levels of the fruits may have a substantial impact on organoleptic properties and human health (Oms-Oliu et al., 2013; Johanningsmeier et al., 2016). In an earlier study, and using a single genotype (Dixiland peach fruits), the metabolomic changes induced by short cold and heat treatment, used to prevent CI, allowed the identification of metabolites which may be involved in priming the fruit to cope with stress situations (Lauxmann et al., 2014). Nonetheless, further metabolomic studies using 15 different peach varieties revealed a great diversity in the content of key metabolites involved in organoleptic properties and protection against stress (Monti et al., 2016). Thus, these studies opened the question on whether it would be possible to associate a metabolic profile of a particular genotype with differential CI susceptibility in peach.

In the present study, a metabolite profiling study after short and long cold storage of six peach varieties with differential susceptibility to develop mealiness (Genero et al., 2016) was performed in order to identify, among the metabolic changes induced by cold, those that may be functionally related to CI resistance in peach. The results indicate that the differential metabolic rearrangements due to cold in peach fruits are related with the CI resistance. Moreover, raffinose levels after long cold treatment quantitatively correlated with the resistance to develop mealiness after subsequent ripening at ambient temperature, emerging as a candidate biomarker of this disorder. The identified metabolic changes that are associated with cold storage may aid in the improvement of peach fruits, with the goal of engineering fruits with higher quality for consumers.

## Materials and methods

### Fruit material and postharvest treatments

**Figure 1:**
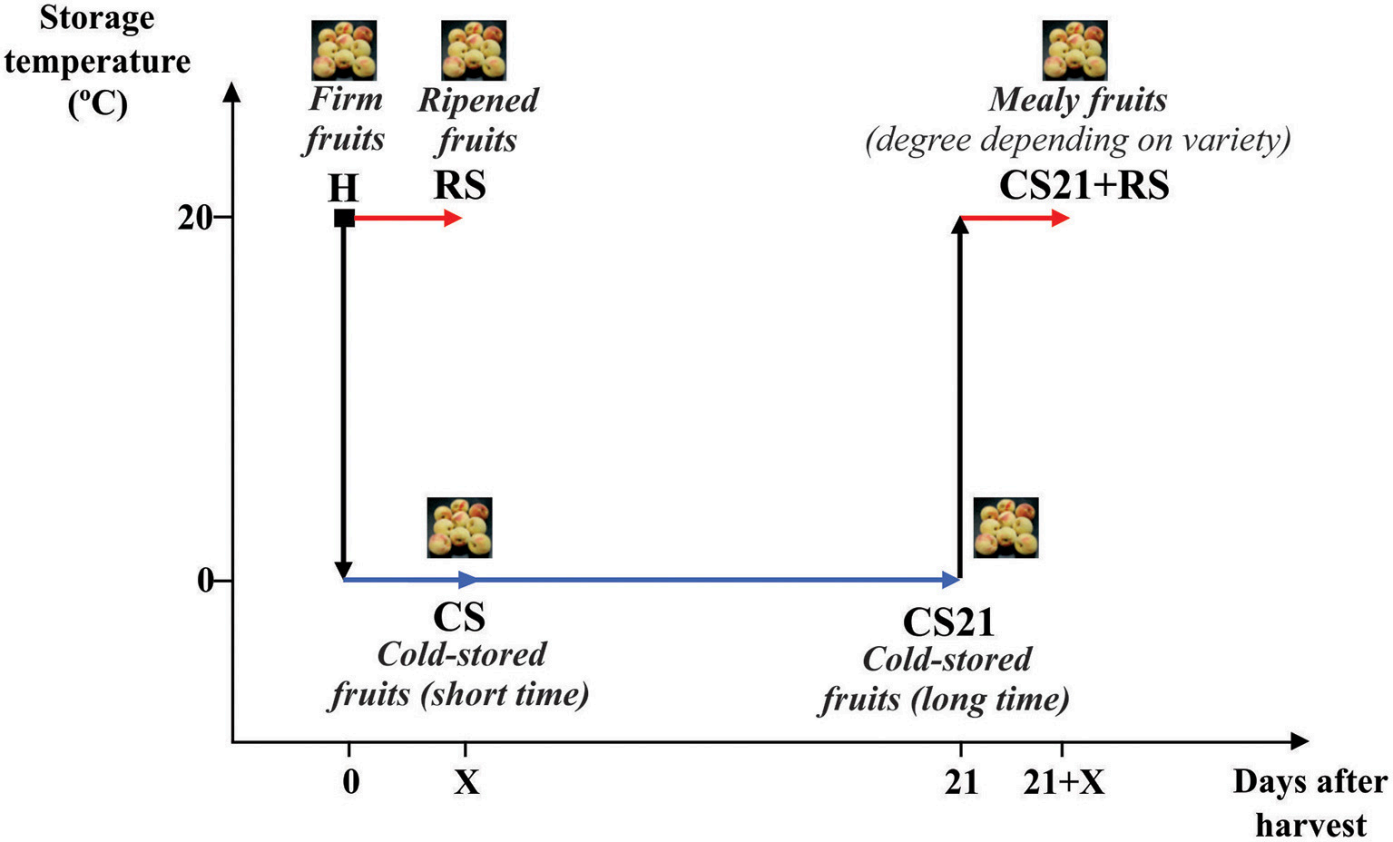
**Schematic representation of peach fruit treatments after harvest**. Peach fruits were harvested at physiological maturity (H, firm fruits) and allowed to ripen at 20°C until reaching organoleptic characteristics suitable for consumption for variable days depending on variety: 3 days for R2, 4 days for SL, or 5 days in the case of FD, EL, RG, and LM. Alternatively, after harvest, fruits were stored at 0°C for 3 (R2), 4 (SL), or 5 (FD, EL, RG, and LM) days (CS); or for 21 days (CS21) followed by 3 (R2), 4 (SL), or 5 (FD, EL, RG, and LM) days at 20°C (CS21+RS).

Assays were conducted with peach (*Prunus persica* L. Batsch) fruit of six different varieties grown in the Estación Experimental Agropecuaria INTA, San Pedro, Argentina. The varieties selected were: Flordaking (FD), Rojo 2 (R2), Springlady (SL), Red Globe (RG), Elegant Lady (EL), and Limón Marelli (LM). The principal agronomic characteristics of each variety are described in Monti et al. (2016) and Table 1. Fruits were collected at S4 stage (Lombardo et al., 2011); with flesh firmness between 40 and 70 N depending on the variety (Monti et al., 2016). Harvested fruits (H) were manually selected for uniformity of color, size and firmness, and divided into four groups (Figure 1). One group, called RS (room temperature-storage fruits), was kept in a chamber at 20°C and 90% relative humidity until reaching firmness and organoleptic characteristics suitable for consumption. The time required for ripeness was dependent on the variety: 3 days in the case of R2, 4 days in the case of SL, and 5 days for FD, RG, EL, and LM. A second group of fruits (CS, cold-storage fruits) was stored at 0°C and 90% relative humidity for 3 (R2), 4 (SL), or 5 (FD, RG, EL, and LM) days. The third group of fruits was stored at 0°C and 90% relative humidity for 21 days (CS21 fruits); while the fourth group was stored at 0°C and 90% relative humidity for 21 days followed by 3 (R2), 4 (SL), or 5 days (for FD, RG, EL, and LM) at 20°C (CS21+RS fruits). Representative mesocarp tissue was collected from at least 20 fruits from the different groups (H, RS, CS, CS21, and CS21+RS; Figure 1), immediately frozen in liquid nitrogen and stored at −80°C for further experiments. The results shown in the present work correspond to fruits collected during the 2009/2010 season, although similar results were obtained for some peach varieties grown during 2011/2012.

**Table 1:** **Principal agronomic characteristics and degree of mealiness resistance of peach fruits**.

**Variety**	**Harvest date**	**Stone adhesion**	**Flesh texture**	**Expressible juice (%)**		**Degree of mealiness resistance**
				**RS**	**CS21+RS**	
Flordaking (FD)	Early	Semi free stone	Melting	62.1 ± 8.3 cd	49.3 ± 9.8 ab	0.79 Susceptible
Rojo 2 (R2)	Early	Semi free stone	Melting	68.7 ± 7.9 d	57.3 ± 11.2 bc	0.83 Intermediate
Springlady (SL)	Early	Free stone	Melting	62.0 ± 6.5 cd	60.6 ± 4.1 cd	0.98 Resistant
Red Globe (RG)	Mid	Free stone	Melting	44.0 ± 23.9 ab	59.9 ± 11.6 bc	1.36 Resistant
Elegant Lady (EL)	Late	Free stone	Melting	58.1 ± 14.6 cd	65.8 ± 4.3 cd	1.13 Resistant
Limón Marelli (LM)	Late	Cling stone	Non-melting	44.7 ± 8.3 ab	41.4 ± 4.6 a	0.93 Resistant

*Depending on the harvest date, the varieties used are classified as early, mid or late varieties (Monti et al., 2016). Firmness and soluble solids of the fruits after different postharvest conditions are indicated in Supplemental Table 1. Expressible juice was measured in peach fruits after ripeness at 20°C (RS) and after cold storage at 0°C for 21 days followed by ripeness at 20°C (CS21+RS). Values represent the mean of five determinations ± SD. Different letters within each parameter indicate statistically significant differences (p < 0.05). The degree of mealiness resistance was calculated as the ratio of expressible juice in CS21+RS relative to RS fruits, depending on which, the varieties were classified as susceptible, intermediate, or resistant to mealiness*.

### Fruit quality trait determination

Flesh firmness and soluble solid content were determined as previously described (Borsani et al., 2009) using about 20–30 fruits from each postharvest treatment group. Fruits from CS21 and CS21+RS groups were visually evaluated for CI symptoms. The wooly texture was evaluated by measuring the amount of expressible juice using from 20 to 30 fruits as described in Lill and van der Mespel (1988). Expressible juice is indicated as the percentage of free juice in total tissue used from each fruit. The degree of mealiness resistance was calculated as the ratio of expressible juice detected in CS21+RS in relation to the one measured in RS fruits. The varieties were classified as resistant to mealiness when the % of expressible juice was not statistically significant different when comparing RS and CS21+RS fruits (SL, RG, EL, and LM; Table 1). In the case of statistically significant differences, the varieties were classified as intermediate (R2, Table 1) or susceptible (FD, Table 1), depending on the difference between these values. Other CI symptoms, as flesh browning or internal reddening, were not detected in the varieties selected and postharvest conditions applied in the present work.

### Metabolite measurements

Metabolite analysis by Gas Chromatography-Mass Spectrometry (GC-MS) was carried out essentially as described by Roessner-Tunali et al. (2003). Mesocarp tissue of peach fruits was ground using ceramic mortar and pestle pre-cooled with liquid nitrogen. Two hundred and fifty milligrams of the powder was used for metabolite extraction using 3 mL of methanol. Internal standard (180 μL, 0.2 mg ribitol mL^−1^ water MiliQ) was subsequently added for quantification purposes. The mixture was extracted for 15 min at 70°C (vortexing every 3 min) and mixed vigorously with pre-cooled water MiliQ (1.5 mL). After centrifugation at 2200 × *g*, an aliquot of the supernatant (50 μL) was transferred to a reaction tube (1.5 mL) and vacuum dried. Tubes were filled with argon gas and stored at −80°C. Samples were derivatized and GC-MS performed as described by Roessner-Tunali et al. (2003). Mass spectra were cross-referenced with those in the Golm Metabolome Database (Kopka et al., 2005). Five independent determinations using three different fruits, and repeated three times each, were performed for each sample analyzed. Metabolite quantification was based on the relative peak response area of each chromatogram and expressed relative to the internal standard (ribitol). The relative values were also expressed as log_2_ using the MultiExperiment Viewer software using a color scale (MeV v4.4.1, http://www.tm4.org/, Saeed et al., 2003). Determination of the absolute concentrations of myo-inositol, galactinol, and raffinose was performed by comparison to calibration standard curve response ratios of various concentrations of standard solutions, including the internal standard ribitol, which were derivatized concomitantly to tissue samples.

### Statistical analysis

Principal component analysis (PCA) performed on data sets obtained from metabolite profiling and Correlation analysis between metabolites based on Pearson correlation were conducted using the software package XLSTAT (Microsoft Excel). The data were log_2_ transformed and normalized to the amount found in EL fruits at harvest. Hierarchical clustering analysis (HCA) was performed using the software package XLSTAT (Microsoft Excel). Regression analysis was performed using SigmaPlot 12.0, Systat Software, Inc. Data presented were analyzed using Two Way Analysis of Variance (ANOVA) with storage temperature and genotype as factors.

## Results

### Postharvest treatments and chilling injury symptoms of peach fruits from six different varieties

Six peach (*P. persica* L. Batsch) varieties with different agronomic characteristics (Table 1; Monti et al., 2016) were selected in the present work: Flordaking (FD), Rojo 2 (R2), Springlady (SL), Red Globe (RG), Elegant Lady (EL), and Limón Marelli (LM). Fruits from each variety were collected at commercial maturity and flesh firmness between 40 and 70 N, which allowed the ending of the ripening process to take place after harvest (Supplemental Table 1). Harvested fruits (H) were stored in chambers at 20°C for 3–5 days depending on the variety, until reaching firmness and organoleptic characteristics suitable for consumption (RS fruits; Figure 1, Supplemental Table 1). Firmness of RS fruits was nearly 10 N, with the exception of LM, which is a non-melting variety (Table 1); and RG, which, although displaying higher firmness (nearly 27 N), organoleptic characteristics indicated a ripened state (Supplemental Table 1). Another group of fruits were stored at 0°C for short time (3–5 days depending on the variety; CS fruits); or for 21 days (CS21 fruits) (Figure 1). The cold storage treatment at 0°C for 21 days was selected because it successfully discriminates peach varieties according to their different degrees of resistance/susceptibility to mealiness (Genero et al., 2016). Firmness of CS and CS21 fruits was similar to the one measured in H fruits for each variety (Supplemental Table 1). After 21 days at 0°C, fruits were stored at 20°C for ripening (CS21+RS fruits; Figure 1). Firmness (between 5 and 27 N) and organoleptic characteristics of CS21+RS fruits were suitable for consumption (Figure 1, Supplemental Table 1). Soluble solid content was measured in fruits from all postharvest conditions: H, RS, CS, CS21, and CS21+RS; with no significant differences detected comparing the different postharvest conditions in practically all the varieties (Figure 1, Supplemental Table 1).

RS and CS21+RS fruits from all varieties displayed firmness and organoleptic properties suitable for consumption; and the visual evaluation of CS21+RS fruits indicated no apparent evidence of CI symptoms in any variety. However, although not externally visible, it is well known that CI symptoms may be present; so, the mealy texture was evaluated by measuring the amount of expressible juice in RS and CS21+RS fruits (Table 1). To obtain a quantitative measure of the resistance to mealiness, we calculated the ratio of expressible juice in CS21+RS relative to RS fruits (Table 1), which gave a value that allowed us to classify the varieties according to their degree of mealiness resistance. In this way, the varieties were classified in: susceptible (FD); intermediate (R2); and resistant to mealiness (EL, LM, RG, and SL) (Table 1). Other CI symptoms, such as flesh browning or internal reddening, were not detected in the varieties selected and the postharvest conditions applied in the present work.

### Metabolite profiles of peach fruits from six varieties subjected to different postharvest treatments

**Figure 2:**
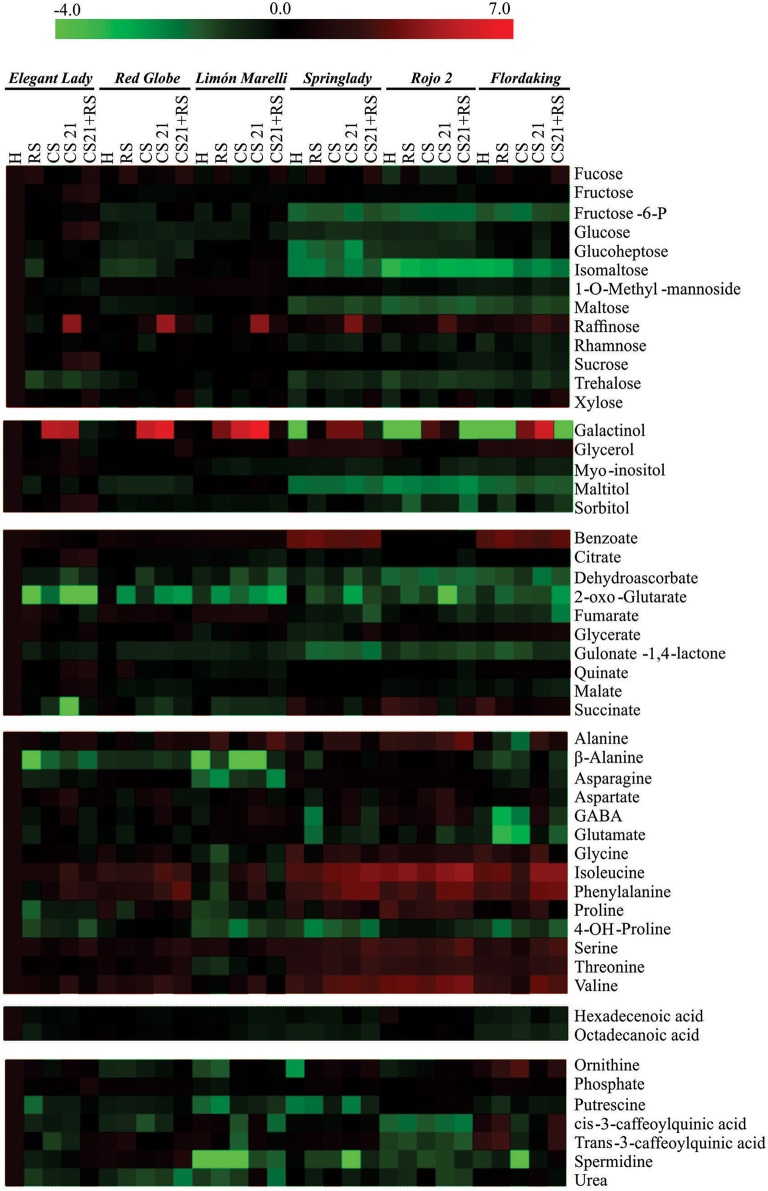
**Distribution of metabolites analyzed by GC-MS in peach fruits during ripening and after cold storage**. The graph shows the relative level of each metabolite to its amount found in harvested Elegant Lady (EL) fruits. Normalized values are shown on a color scale (shown at the top of the figure), which is proportional to the content of each metabolite. Mean values of five independent determinations for each sample were normalized to harvested EL fruits and expressed as log_2_ using the MultiExperiment Viewer software (MeV v4.4.1, Saeed et al., 2003). The relative metabolite levels and standard errors of each peach sample are shown in Supplemental Table 2.

Changes in metabolite levels associated to different postharvest treatments in the mesocarp of fruits from the six varieties were assessed by GC-MS. By this technique, 51 polar metabolites were monitored, and their levels were expressed relative to the internal standard ribitol. In order to easily compare the changes in the levels of the 51 metabolites detected, the content of each metabolite in FD, R2, SL, RG, EL, and LM peach fruits in the five postharvest conditions (H, RS, CS, CS21, and CS21+RS); were expressed relative to the levels of the metabolites detected in EL fruits at harvest, which was arbitrarily selected (Supplemental Table 2). The identified metabolites were divided into sugars (13), sugar alcohols (5), organic acids (10), amino acids (14), fatty acids (2), and miscellaneous compounds (7). In order to easily visualize the metabolic differences among the varieties and after different postharvest conditions, the normalized relative levels of each metabolite are shown in Figure 2 using a color scale, which is proportional to the content of each identified metabolite. A large degree of metabolic variation, depending on both the variety and the postharvest treatment, is clearly observed (Figure 2).

### Hierarchical clustering analysis (HCA) and principal component analysis (PCA) of metabolic data

The resultant metabolic complements of the six peach varieties subjected to different postharvest treatments were then compared with each other using HCA and PCA.

**Figure 3:**
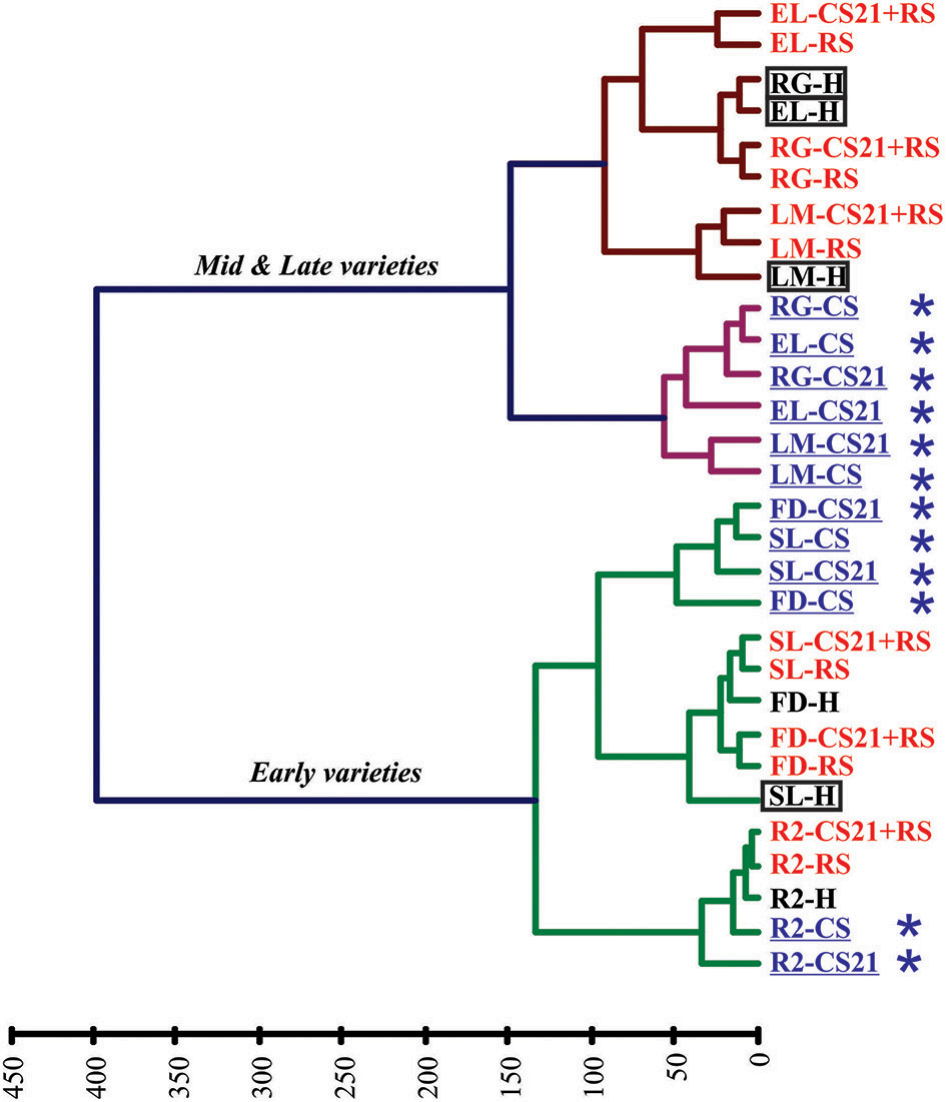
**Hierarchical clustering analysis (HCA) of peach fruits from six varieties subjected to different postharvest treatments depending on metabolic profile**. Peach fruit samples from six varieties (FD, R2, SL, RG, EL, and LM) subjected to different postharvest treatments (H, RS, CS, CS21, and CS21+RS) are divided in two principal clusters depending on harvest time (early vs. mid & late varieties). Further divisions are dependent on the variety or the particular postharvest treatment. Cold-treated samples are indicated in blue and with an asterisk (^*^), while ripened samples (RS) are indicated in red. Resistant to mealiness varieties (LM, SL, RG, and EL, Table 1) are marked with a rectangle.

Applying HCA to the full data set obtained following GC-MS analysis of the different peach samples revealed interesting results (Figure 3). The samples were separated into two main clusters, which divide the peach fruits depending on the harvest time of the varieties: early vs. mid & late varieties (Table 1, Figure 3). The mid & late varieties group was further subdivided into two different subgroups, one of which is composed by fruits that were subjected to either short or long cold storage treatment (CS and CS21 fruits); and the other it is composed by H and ripened fruits (Figure 3). Within this last subgroup, and independently to whether the fruits were subjected to 21 days of cold storage or not, ripened fruits of each variety (RS and CS21+RS) cluster together and separate from H fruits (Figure 3). Regarding the early varieties, two groups that are variety-dependent, can be found: R2 in one main branch, and FD and SL in another (Figure 3). For each sub-cluster, cold-stored samples for short, and long time periods cluster together (CS and CS21 samples); while ripened samples (RS and CS21+RS), independently to whether they were subjected to cold storage or not prior to ripeness, group together (Figure 3). On the other hand, the HCA map obtained indicates that the peach varieties resistant to mealiness (LM, SL, RG, and EL; Table 1) are distributed in the two different main clusters (in both early and mid & late varieties), with no obvious particular common metabolic pattern evident by this grouping technique (Figure 3).

**Figure 4:**
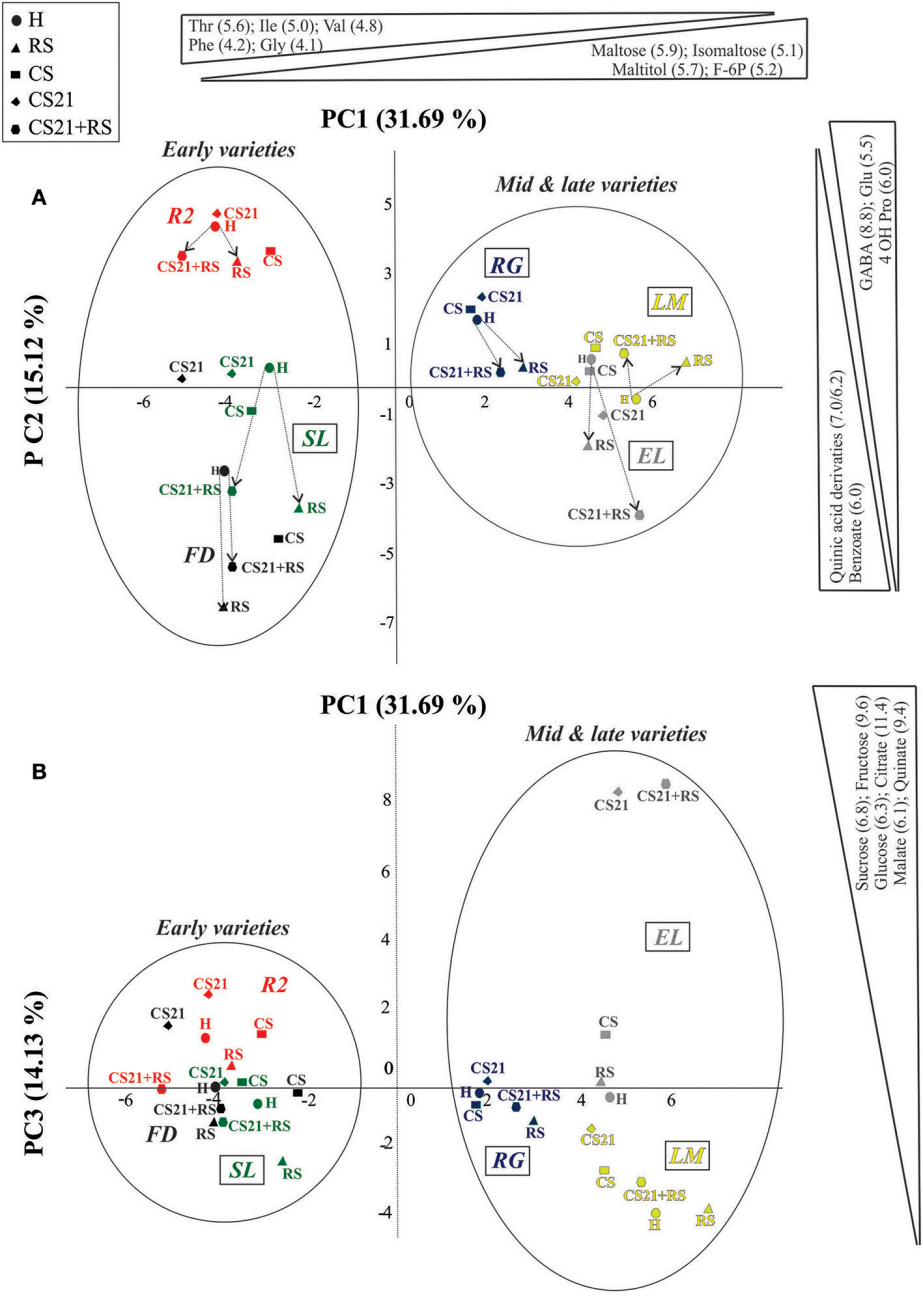
**Principal component analysis (PCA) of the metabolic profile of peach fruit varieties subjected to different postharvest treatments**. Metabolic profile of peach fruit samples from six varieties (FD, R2, SL, RG, EL, and LM) subjected to different postharvest treatments (H, RS, CS, CS21, and CS21+RS) was analyzed by PCA. The first principal component (PC1) is shown on the *x*-axis vs. the second principal component (PC2) on the *y*-axis in **(A)**; and vs. the third principal component (PC3) on the *y*-axis in **(B)**. Early and mid & late varieties, which are clearly separated by PC1, are indicated with circles. The metabolites that contribute the most to each component separations are indicated on the top (PC1) and on the right (PC2 in **A** and PC3 in **B**). The variance explained by each component (%) is indicated in parentheses. In **(A)**, the path from H to ripened fruits (RS and CS21+RS) is indicated by arrows. Resistant to mealiness varieties (LM, SL, RG, and EL, Table 1) are marked with a rectangle.

PCA revealed also interesting grouping of the samples (Figure 4). The first principal component (PC1, 31.69% of the variance) separates the samples depending on harvest time, with mid & late varieties on the positive and early varieties on the negative side (Figure 4). Among the metabolites that most contribute to PC1 separation, higher levels of maltose, isomaltose, maltitol, and fructose 6-P are found in mid & late varieties; while higher levels of Thr, Ile, Val, Phe, and Gly are found in early varieties (Figure 4, Supplemental Table 3). Within early varieties, PC2 (15.12% of the variance) is able to clearly discriminate R2 (Figure 4), which also clusters separately in the HCA grouping (Figure 3). Among the metabolites that contribute the most to PC2 separation, higher levels of GABA, 4OH Pro, and Glu are found in R2; while higher levels of benzoate and quinic acid derivatives in FD (Figure 4, Supplemental Table 3). From this graph, a common path is observed when going from H to ripened fruits (RS and CS21+RS; Figure 4). This path involves movements to the negative side of PC2, with the exception of LM, the only non-melting variety analyzed, in which the path goes to the positive side (Table 1, Figure 4). On the other hand, PC3 (14.13% of the variance) is able to discriminate mid & late varieties more than early varieties (Figure 4). Particularly, a clear separation of cold treated EL fruits for 21 days (CS21 and CS21+RS) can be observed, which are samples characterized by a large increase in sugars like sucrose, glucose and fructose, and organic acids such as citrate, malate, and quinate (Figure 4, Supplemental Table 3). Again, as observed with HCA clustering, varieties resistant to mealiness (LM, EL, RG, and SL) can be found in different parts of the PCA graphs (Figure 4).

### Cold-metabolic rearrangements in peach fruits from six different varieties

**Figure 5:**
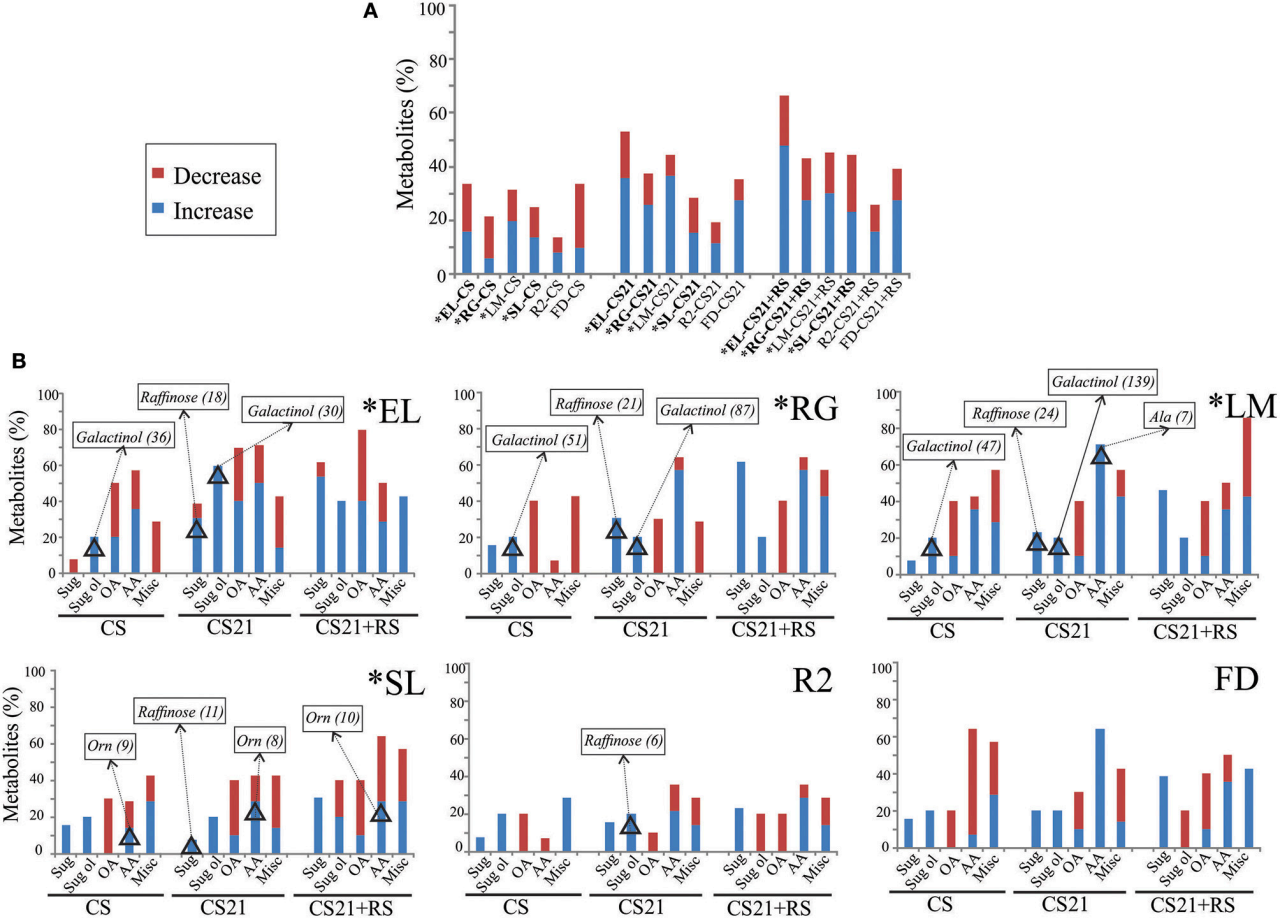
**Effect of cold-treatments on the metabolome of peach fruits from different varieties. (A)** For each peach fruit variety (EL, RG, LM, SL, R2, and FD), metabolite levels in CS, CS21, and CS21+RS samples were compared with those detected at H, and the % of metabolites that are significantly increased or decreased are shown. **(B)** For each variety (EL, RG, LM, SL, R2, and FD), the % of sugars (Sug), sugars alcohol (Sug ol), organic acids (OA), amino acids (AA), and miscellaneous compounds (Misc); that are significantly increased or decreased in CS, CS21, and CS21+RS samples relative to H are shown. In each graph, the metabolites that resulted modified more than 5 times are shown in rectangles, followed by the ratio of modification relative to H in parenthesis. The metabolites that are modified from or to *not detectable* levels are not highlighted in this graph, but they were considered in the number of metabolites that are increased or decreased. The asterisk denotes varieties that are resistant to chilling injury (Table 1). The relative metabolite levels of each peach sample are shown in Supplemental Table 4.

To analyze the extent to which cold treatment modifies the metabolic content of peach fruits, metabolite levels in CS, CS21, and CS21+RS samples were compared with those detected at harvest (H) in each peach varieties (Figure 5). Figure 5A shows the % of metabolites that are significantly modified, either increased or decreased, in CS, CS21, and CS21+RS, for each peach fruit variety. A general overview of the metabolic rearrangements of peach fruits after each cold treatment shows that the metabolic alterations are dependent on both the variety and the time of exposure to cold. From Figure 5A, it is also evident that, in CS samples and for the majority of the varieties, with the exception of LM, the number of metabolites that are decreased are higher (in EL, RG, and FD), or equal (in SL and R2), than those that are increased (Figure 5A, Supplemental Table 4). In contrast, for both CS21 and CS21+RS from all the varieties, the number of metabolites that is increased is much larger than the number that is decreased (Supplemental Table 4, Figure 5A). The increase/decrease ratios vary from 1.4 to 2.6 depending on the variety. This indicates that cold treatment of peach fruits results in an early global response of decrease of metabolite content at CS, followed by a response of increase of particular metabolite levels at CS21 and CS21+RS. From Figure 5A, it is also obvious that, among all varieties, the metabolome of R2 is the one that is less reconfigured due to cold; while the metabolome of EL is the one that is most highly modified, especially after long cold treatment (Figure 5A, Supplemental Table 4).

On the other hand, cold treatment of peach fruit causes an increase in the level of certain sugars; typically raffinose, and depending on the variety glucoheptose, isomaltose, and/or trehalose (Figure 5B, Supplemental Table 4). The only sugar that exhibited decreased levels in response to cold is trehalose in EL (Figure 5B, Supplemental Table 4). In particular, short cold treatment (CS) induces the increase of just one or two sugars in each variety; while longer cold treatment (CS21) involves increases in a larger number of sugars and, to a greater extent, in raffinose levels in all the varieties (from 3- to 24-times; Figure 5B, Supplemental Table 4). In addition, in CS21+RS, sugars are more dramatically altered, including typical modifications detected during ripening, such as for example an increase in fucose levels across all the varieties (Monti et al., 2016). Regarding sugar alcohols, in addition to variety-specific modifications in sorbitol levels, the cold response of peach fruits is characterized by dramatic modifications in galactinol levels, which increases up to 140 fold or from undetectable levels after short and long cold treatments (Figure 5B, Supplemental Table 4). Organic acid levels modifications in peach fruits after cold include a profound decrease of succinate and 2-oxo-glutarate in all varieties irrespective of the length of cold treatment (Figure 5B, Supplemental Table 4). Modifications in the level of other organic acids are more variety-dependent, with increases in glycerate in EL, LM, SL, and FD; decreases in dehydroascorbate in EL, RG, and LM; and rises in citrate, malate, and quinate in EL (Figure 5B, Supplemental Table 4). Although amino acid levels are drastically reconfigured after cold in all the varieties, these modifications are strictly dependant on the variety, with no obvious common pattern of response to cold (Figure 5B, Supplemental Table 4). Ornithine levels are increased in all the varieties, at least in one condition after cold treatment, with drastic increases up to 10 fold in SL (Figure 5B, Supplemental Table 4). Changes in the levels of quinic acid derivatives, putrescine, urea, and spermidine are dependent on both the variety and treatment considered (Figure 5B, Supplemental Table 4).

### Metabolite-metabolite correlation analysis in the six peach varieties

Correlation analysis performed on the entire data set of metabolites of each of the six different varieties at the different postharvest treatments were performed in order to identify associations of metabolites and a more detailed evaluation of the behavior of the metabolite network (Supplemental Figure 1). Out of 1275 pairs of metabolites analyzed, only between 97 and 140 metabolite-metabolite correlations were significant (*p* < 0.05). The number of positive vs. negative correlations varied depending on the variety (Supplemental Table 5). EL and RG displayed nearly 3 times more positive than negative metabolite-metabolite correlations. LM, FD, and SL displayed between 2.1 and 1.7 higher positive than negative correlations, while in R2 negative and positive metabolite correlations were essentially equivalent (Supplemental Table 5).

In general, the metabolite-metabolite correlations detected in the present work were highly dependent on the variety considered; however, some particular behaviors of the metabolic network are worth mentioning. For example, it is remarkable that in the most susceptible varieties (FD and R2), negative correlations between sucrose and glucose and some amino acids such as Ile, Phe, Val, Glu, and Asn, are evident; yet these were not detected in the resistant varieties (Supplemental Figure 1). Moreover, in some varieties resistant to mealiness, some positive correlations between sugars and amino acids are detected, e.g., glucose correlates positively with Asp, Ile, Phe, and Val in LM (Supplemental Figure 1). In addition, raffinose positively correlates with several amino acids, such as GABA, Pro, Val, Ile, and Asp in all genotypes, with the exception of SL (Supplemental Figure 1). Xylose correlates negatively to several organic acids such as malate, fumarate, and citrate, in the early varieties (FD, R2, and SL); but not in mid & late varieties (Supplemental Figure 1). Finally, in EL, positive correlations between glucose and fructose with sorbitol and organic acids like malate and citrate were detected (Supplemental Figure 1). Thus, these metabolic associations could be useful to identify variety-specific and conserved co-regulated pathways and biochemical regulatory mechanisms in the peach fruit.

### Correlation of metabolite content after cold treatment to the degree of mealiness resistance

The modification of raffinose and galactinol levels in all the peach fruit varieties is remarkable among the changes in the relative levels of metabolites due to cold treatment (Figure 5B, Supplemental Table 4). Thus, the concentration of these compounds, along with *myo*-inositol, which participates in raffinose metabolism, was quantified following different postharvest treatments (Table 2). At harvest, the raffinose level ranges from 1.1 (EL) to 1.8 (FD) μg/gFW in the six peach varieties; and these levels are not significantly modified in RS (Table 2). However, cold treatment induces a drastic increase in raffinose levels in a cold exposure-dependent manner. Dramatic increases, from 5.4 (FD) to 30.3 (RG) fold relative to the levels detected at harvest, were detected in CS21 (Table 2). Galactinol was not detected at harvest (H) and after ripening (RS) in any of the varieties with the exception of LM (Table 2). Cold treatment induces increases in galactinol levels in peach fruits; reaching from 0.5 (R2) to 2.3 (LM) μg/gFW at CS21 in the six peach varieties (Table 2). Therefore, although galactinol levels do respond to cold treatment, their levels are largely invariant across the different varieties. The level of *myo*-inositol varies from 8.1 (R2-CS21+RS) to 15.8 (EL-RS) μg/gFW in all the samples analyzed, with no particular response to cold treatment or variation across the varieties (Table 2).

**Table 2:** **Quantitative determination (μg/g FW) of myo-inositol, galactinol, and raffinose**.

**Sample**	**Metabolite (**μ**g/g FW)**		
	**Myo-inositol**	**Galactinol**	**Raffinose**
FD-H	10.8 ± 0.8 bcdef	nd	1.8 ± 0.3 a
FD-RS	10.2 ± 0.3 abcd	nd	2.0 ± 0.1 a
FD-CS	11.9 ± 0.4 cdefg	0.6 ± 0.0 ab	2.9 ± 0.4 a
FD-CS21	9.7 ± 0.7 abc	1.2 ± 0.3 c	9.7 ± 2.9 b
FD-CS21+RS	10.1 ± 0.4 abcd	nd	3.1 ± 0.2 a
R2-H	10.9 ± 1.1 bcd	nd	1.3 ± 0.1 a
R2-RS	11.1 ± 0.9 bcdef	nd	1.4 ± 0.1 a
R2-CS	12.0 ± 0.9 cdefg	0.5 ± 0.0 a	1.8 ± 0.2 a
R2-CS21	8.8 ± 0.5 ab	0.5 ± 0.1 a	12.0 ± 2.0 b
R2-CS21+RS	8.1 ± 0.8 a	nd	2.1 ± 0.2 a
SL-H	12.8 ± 0.9 cfgh	nd	1.3 ± 0.2 a
SL-RS	13.3 ± 0.8 fghi	nd	1.7 ± 0.1 a
SL-CS	12.0 ± 1.0 cdefg	0.6 ± 0.1 a	3.0 ± 0.2 a
SL-CS21	10.9 ± 0.3 bcd	0.6 ± 0.0 a	25.0 ± 1.4 c
SL-CS21+RS	10.5 ± 1.0 abcd	nd	2.4 ± 01 a
RG-H	12.0 ± 0.7 cdefg	nd	1.3 ± 0.1 a
RG-RS	15.0 ± 1.0 hij	nd	1.7 ± 0.1 a
RG-CS	13.7 ± 0.9 ghij	0.7 ± 0.2 ab	3.6 ± 0.6 a
RG-CS21	12.0 ± 0.6 cfghi	1.7 ± 0.3 d	39.5 ± 0.7 e
RG-CS21+RS	13.4 ± 1.1 fghi	nd	2.9 ± 0.4 a
EL-H	14.0 ± 0.2 ghij	nd	1.1 ± 0.1 a
EL-RS	15.8 ± 0.9 j	nd	1.5 ± 0.1 a
EL-CS	15.0 ± 1.0 ghij	0.9 ± 0.1 abc	2.3 ± 0.2 a
EL-CS21	14.8 ± 1.2 ij	0.8 ± 0.1 abc	26.0 ± 3.0 c
EL-CS21+RS	12.3 ± 0.5 defgh	nd	1.7 ± 0.1 a
LM-H	11.2 ± 0.7 cdef	0.5 ± 0.0 a	1.2 ± 0.0 a
LM-RS	9.9 ± 0.6 abcd	0.6 ± 0.0 a	1.7 ± 0.3 a
LM-CS	9.8 ± 0.9 abc	1.1 ± 0.1 bc	2.5 ± 0.2 a
LM-CS21	11.0 ± 0.8 bcd	2.3 ± 0.3 e	31.3 ± 1.4 d
LM-CS21+RS	10.4 ± 0.5 abcd	0.5 ± 0.0 a	2.6 ± 0.2 a

*Values (± SD) represent the mean of five independent determinations with three technical repetitions each one. All metabolites were quantified by GC-MS using calibration curves, which were run in parallel. nd: not detected. Different letters within each parameter indicate statistically significant differences (p < 0.05)*.

**Figure 6:**
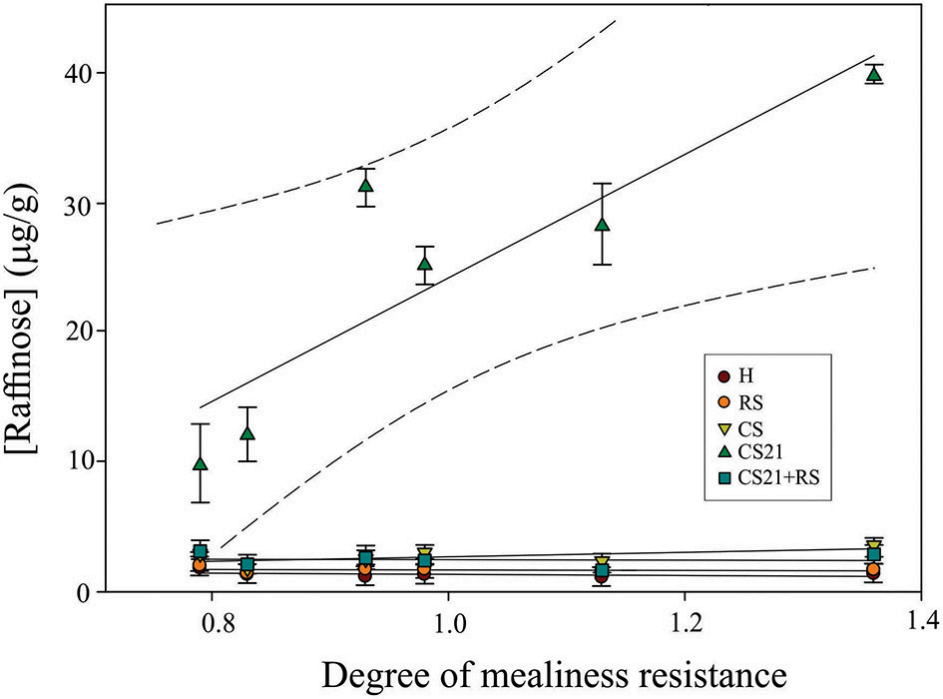
**Linear regression line fit between the degree of mealiness resistance and raffinose concentration at harvest and after different postharvest treatments of peach fruit**. Multiple regression analysis was performed between raffinose concentration at H; RS; CS; CS21; or CS21+RS, and the degree of mealiness resistance of the six peach varieties (Table 1). The following regression coefficients were obtained for each postharvest condition: H (*R*^2^ = 0.13); RS (*R*^2^ = 0.038); CS (*R*^2^ = 0.35); CS21 (*R*^2^ = 0.78); and CS21+RS (*R*^2^ = 0.0053). The 95% confident intervals are indicated in the case of CS21.

One of the goals of the present work was to identify metabolic markers that could be correlated to mealiness resistance in peach fruits. In this way, a regression analysis between the degree of mealiness resistance (Table 1) and the level of raffinose, the metabolite that seems to most clearly discriminate resistance to mealiness across the varieties, was performed. In the multiple regression analysis approach using raffinose level at harvest and at the different postharvest treatments, only raffinose concentration after long cold treatment (CS21) displayed a significant regression value (*R*^2^ = 0.78; Figure 6) with the degree of mealiness resistance of the six peach varieties. No significant correlation between the degree of mealiness resistance and raffinose levels at harvest (*R*^2^ = 0.14), after ripening at 20°C (RS; *R*^2^ = 0.038); after short cold treatment (CS; *R*^2^ = 0.035); or after ripening following long cold storage (CS21+RS; *R*^2^ = 0.0053) was detected (Figure 6).

## Discussion

### Harvest time, cold treatment, and ripening: key factors involved in defining the metabolic status of peach fruits

HCA and PCA analysis of the metabolite profiles of peach fruits from the six varieties selected in the present work indicated that the most relevant factors involved in defining the overall metabolic status of the peach fruits are: (1) Harvest time; (2) Cold treatment; and (3) Ripening (Figures 3, 4). The first level of separation of the peach fruits is related to the harvest time, as was previously observed using a larger number of varieties (Monti et al., 2016). The second level of separation is a bit different depending on the harvest time, although it is clearly related to cold treatment and the ripening process in both groups (Figure 3). Among mid & late varieties, fruits subjected to cold treatment (CS and CS21) cluster separately from harvest (H) and ripened fruits (RS and CS21+RS) (Figure 3). Several important conclusions can be derived from this interesting clustering. First, that cold, regardless the duration of exposure, induces a novel and similar metabolic status in the fruits from this group; whilst the fact that cold-stored EL, RG, and LM group together indicates that this is independent of the particular genotype considered. Secondly, it is clear that the ripening process superimposes a metabolic reconfiguration over that induced by cold, as in all six varieties analyzed RS and CS21+RS samples from the same variety are grouped together (Figure 3). Thus, cold stored fruits for 21 days are able to shift to a metabolic status similar to that of RS fruits, indicating that the ripening process, halted by cold, can resume after cold storage following a similar metabolic program.

In relation to the clustering of the early varieties, R2 forms a distinct group, indicating that in this case the genotype seems to be relevant in defining the metabolic status. In this case, as observed for mid & late varieties, ripened R2 samples (RS and CS21+RS) are grouped together. Within the second group of early varieties, comprising FD and SL, metabolic changes due to ripening were superimposed on those occurred after 21 days of cold, rendering similar metabolic states for RS and CS21+RS (Figure 3). Interestingly, in this last group, a mealiness resistant (SL) and a susceptible variety (FD) are found together, suggesting that the overall metabolic status of the fruits is not related to the degree of CI resistance, although the detailed analysis of the metabolic reconfiguration after cold treatment may reveal specific metabolic processes associated with this differential phenotype, as discussed below.

**Figure 7:**
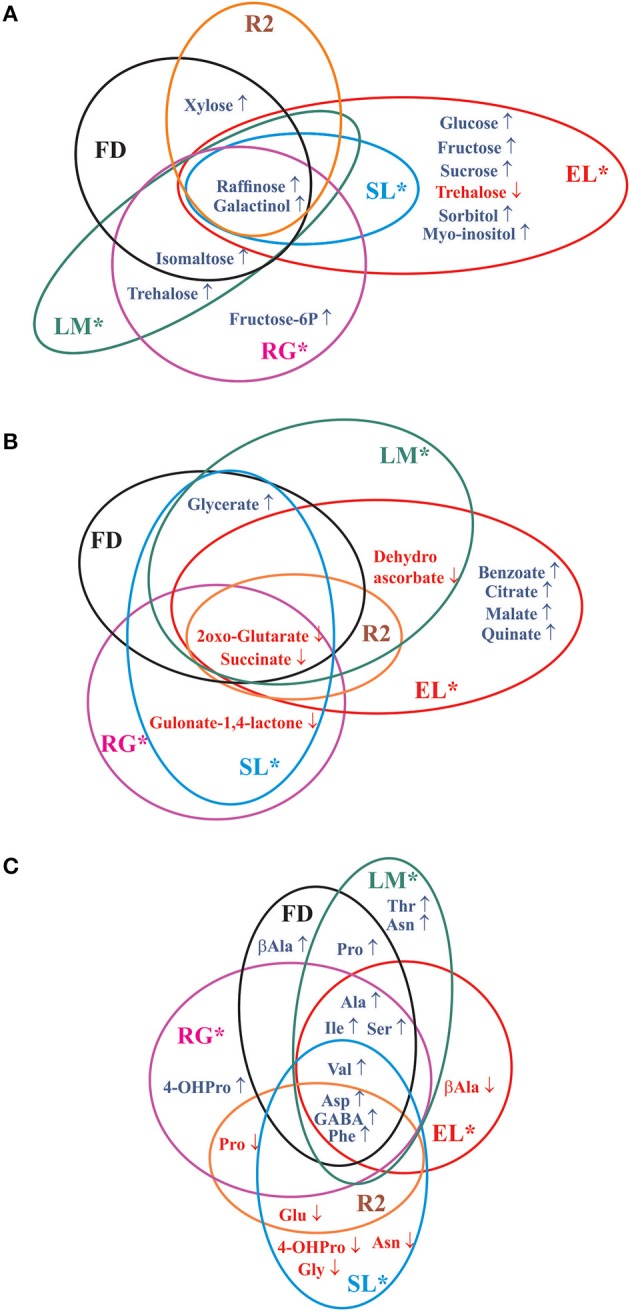
**Commonalities and differences of the metabolic reconfiguration after long cold treatment of the six peach fruit varieties**. The Venn diagrams show significant modifications in sugars and sugar alcohols **(A)**, organic acids **(B)**, and amino acids **(C)** after 21 days of cold storage in the six peach fruit varieties with respect to metabolite levels at harvest (Supplemental Table 4). The asterisk denotes varieties that are resistant to chilling injury (Table 1).

The grouping of peach fruits by PCA leads to similar conclusions as those reached by HCA. Interestingly, PC3 (14.13% of the variance) is able to clearly discriminate EL cold treated fruits (CS, CS21, and CS+21), because of the significantly higher levels of sugars such as sucrose, glucose, and fructose; and organic acids such as citrate, malate, and quinate (Figure 4B). This interesting observation warrants further studies, since EL is the only variety in which this type of cold induced reconfiguration is observed (Figures 7A,B). The identification of the molecular basis of the particular response of EL to cold is a future challenge, since it could aid in defining strategies for the improvement of the organoleptic quality of peach fruits by increasing sugar and organic acids levels while fruits are stored at low temperatures (Cirilli et al., 2016).

### Do the peach fruits from different varieties follow a similar metabolic reconfiguration after cold treatment?

Comparing short and long cold treatment, a higher number of metabolites tend to decrease, rather than to increase, in CS; in contrast to the higher number of metabolites that are increased, rather than decreased, in CS21 (Figure 5A). The first obvious direct effect of cold treatment on metabolite levels may be related to a decrease of the activity of the enzymes already present in the fruit; however, a general decrease of enzyme activity is not necessarily correlated to a decrease in metabolite levels. In this sense, it has been clearly shown that cold regulation of transcripts in Arabidopsis occurs in waves, with some groups of genes showing very early regulation after transfer to cold and others responding much more slowly; while others being transiently modified (Thomashow, 2010; Knight and Knight, 2012). So, the differential effect observed after short and long cold treatment may in fact reflect time differential transcriptional and/or posttranscriptional response to cold, indicating an active reconfiguration of the peach fruit metabolome depending on the time exposed to cold. Indeed, it must be considered that metabolism is not a passive target of cold, because metabolic changes may in turn regulate cold signaling and gene expression, as well as modify the activity of central enzymes allosterically; issues that contribute to different metabolic reconfiguration depending on the time of exposure to cold.

The metabolic response to cold has been extensively analyzed in Arabidopsis, where, as well as in other plant species, a complex reprogramming of the central carbohydrate metabolism was observed. From these studies, it is widely accepted that sucrose, fructose and glucose accumulation is a general response to low temperature stress (Klotke et al., 2004; Korn et al., 2008; Nagele et al., 2012; Wang et al., 2013; Tarkowski and Van den Ende, 2015). Other key sugars and sugar alcohols involved in cold response have also been identified, with galactinol, raffinose, and trehalose being the most studied to date (Livingston et al., 2009; Knaupp et al., 2011; Nagele and Heyer, 2013). Sugars can serve as osmoprotectants of biological membranes and can stabilize macromolecular structures; also it was suggested that sucrose serves as a substrate for other low temperature-induced metabolic alterations. Other metabolites involved in cold response include Pro and GABA, which also share compatible solute-like properties (Bouche and Fromm, 2004; Obata and Fernie, 2012). Despite the existence of widely cold-responsive metabolites, the comparison of the response of different species indicated that specific cold acclimation processes also exist (Hannah et al., 2006; Dauwe et al., 2012; Rohloff et al., 2012; Zuther et al., 2012; Benina et al., 2013).

Here, both common and distinct metabolic reconfigurations due to cold were detected among the varieties analyzed (Figures 5B, 7, Supplemental Table 4). Common changes in sugars and sugar alcohols include a drastic rise in galactinol and raffinose; while other changes are genotype-dependent (Figure 7A, Supplemental Table 4). Regarding organic acids, a dramatic decrease of both 2-oxo-glutarate and succinate levels was notable among all the peach fruit varieties after long cold treatment (Figure 7B, Supplemental Table 4). However, a decrease of 2-oxo-glutarate in all varieties, and of succinate in some varieties (SL, R2, FD, and LM), is also observed in RS samples (Supplemental Table 2). So, this decrease may represent a metabolization, not strictly dependent on cold treatment, of these organic acids after harvest. Both common and differential changes were also detected in the reconfiguration of the amino acid levels following cold storage. GABA, Asp, and Phe increase following long cold treatment in all six peach fruit varieties; while the other amino acid modifications are more genotype-dependent; in relation to the complex pivotal roles of amino acids in plants, for protein biosynthesis, as building blocks for several other biosynthetic pathways, in signaling processes, and in stress responses (Hildebrandt et al., 2015).

Collectively, the diverse array of sugars, sugar alcohols, organic acids, and amino acids modified after cold treatment (Figure 7) constitute peach fruit main metabolic defense mechanisms against cold; metabolic defense, which is successful, in some genotypes, to avoid the appearance of CI symptoms, as discussed below.

### Particular metabolic adjustments can be correlated to mealiness: the case of raffinose levels after 21 days of cold treatment

The results presented here indicate a major restructuration of peach fruit metabolism following exposure to cold; however, metabolite changes may represent a consequence of cold stress without any functional role in peach fruit protection against CI. In order to discriminate the processes that are critical for CI tolerance from those that are merely responsive to low temperature, the metabolic reconfiguration was compared to the degree of mealiness resistance of each genotype (Table 1). HCA and PCA studies revealed no discrimination of peach fruits resistant to mealiness from those that are sensitive to this disorder (Figures 3, 4). This fact indicates that the resistance to mealiness is not characteristic of a particular metabolic group in peach fruit; thus, CI resistance may in fact be more related to differential reconfiguration after cold exposure.

Although a direct correlation of a multigenic trait such as CI tolerance with the concentration of only one metabolite would not be expected, the degree of mealiness resistance of the peach fruit varieties correlated very well to the level of raffinose following 21 days of cold storage (Figure 6). Several studies have confirmed that sugars such as raffinose have the capacity to stabilize membranes by inserting into the lipid head group region of the membranes, helping to prevent leakage when water is removed, as occurs, for example, under low temperatures (Strauss and Hauser, 1986; Vereyken et al., 2001; Hincha et al., 2003; Livingston et al., 2009). Moreover, other studies have indicated that the principal role of raffinose as membrane protectant is performed in fact in the chloroplasts, specifically protecting photosystems (Schneider and Keller, 2009; Knaupp et al., 2011; Nagele and Heyer, 2013). Although it would not be expected that the major protective role of raffinose is performed in plastids in peach fruits, it seems that the steady state levels of raffinose reached after 21 days of cold storage plays a crucial role in protecting peach fruit membranes and, thus, avoiding the appearance of mealiness when peach fruits are transferred to ambient temperatures to ripen. In previous studies, correlation of galactinol content to cold tolerance in strawberry plants was found (Davik et al., 2013).

Another common response to cold of peach fruits from different genotypes is an increase of certain amino acids such as Phe, Asp and GABA (Figure 7). However, the increase of these amino acids is not as dramatic as that of raffinose or galactinol. Moreover, the ratio of amino acids increase is not strictly associated to the degree of mealiness resistance, as for example the highest increase of GABA takes place in FD, the susceptible genotype (Supplemental Tables 2, 4). Thus, the increase in these metabolites may represent a cold response with no strict functional relevance in CI protection; or, alternatively, these metabolites could contribute to the low temperature defense in both CI resistant and susceptible peach fruits, without discriminating the degree of mealiness resistance of the genotypes.

Some amino acids are known to contribute to the tolerance to abiotic stresses; among which, Pro is one of the well-documented osmoprotectants (Obata and Fernie, 2012). However, Pro is induced by cold in the susceptible (FD) peach variety and in only one resistant genotype (LM), indicating that in peach fruits Pro has not a key functional role in preventing mealiness. In addition, depending on the genotype, it appears that some varieties may take advantage of specific reconfigurations for adapting to cold. In this sense, unique features of the reconfiguration of EL metabolome are highly interesting (Figure 7). These modifications may constitute a redundant cellular protection system, where the accumulation of a large variety of metabolites is likely to contribute to the establishment of a robust system to cope with cold. It is worth mentioning that in a previous study, higher levels of sucrose in one peach variety, was linked to enhanced CI tolerance (Wang et al., 2013). Finally, all the other specific metabolic modifications operating in the individual resistant genotypes (Figure 7) may imply unique strategies to counteract low temperature stress, in addition to the global response of increasing raffinose and galactinol levels.

Interestingly, xylose is only increased following 21 days of cold treatment in FD and R2 the most mealiness susceptible genotypes (Table 1, Figure 7). Considering that xylose is a central constituent of hemicellulose of the plant cell walls, its increase in FD and R2, but not in the four varieties resistant to mealiness, may indicate a particular reconfiguration of the cell wall in the most susceptible varieties while being cold-stored for long period of times. This differential cell wall-structure change between resistant and susceptible peach fruits may contribute to the further abnormal degradation of the cell wall pectins when the fruits are allowed to ripen at room temperature, which results in mealiness development in the case of susceptible genotypes (Brummell et al., 2004; Fruk et al., 2014). A possible role of β-xylosidase in the tolerance to CI has been previously suggested by different works (Falara et al., 2011; Genero et al., 2016); and the link of β-xylosidase activity and the level of xylose during cold storage deserves further studies. Regarding the most susceptible genotype (FD) it is also notably the high levels of quinic acids in RS and CS21+RS (Figure 2, Supplemental Table 2), it remains to be analyzed if this affects ripening after cold storage.

Finally, when comparing the metabolomics response to cold of different peach fruit genotypes studied in this work with previous transcriptomic and proteomic studies (Pons et al., 2014, 2015; Almeida et al., 2016), clear differences in the behavior of the metabolome can be detected. In the case of transcriptomic and proteomic studies, it was concluded that alternative cold responses are acting in peach CI tolerant genotypes when compared to susceptible ones; and that these differential responses are active prior to cold exposure. However, in the present work, it was observed that the metabolic status of peach fruits prior to cold treatment is not necessarily correlated to the susceptibility to develop mealiness; moreover, the cold-induced metabolic reconfiguration is more related to the differential susceptibility. Nonetheless, in agreement with our studies, transcriptomic studies identified different transcripts encoding putative galactinol synthases (galactinol is the precursor of raffinose), as part of the cold response in peach fruits with differential susceptibility to CI (Vizoso et al., 2009; Dagar et al., 2013; Pons et al., 2014). Overall, the results presented here show, once again, the tremendous plasticity of the metabolism in plants; and it is therefore concluded that differential metabolic capacities among different peach genotypes determine, at least in part, their ability to cope with changes in environmental conditions.

## Concluding remarks

Considering the results obtained in the present work, it is concluded that the widespread usage of cold storage to preserve fleshy fruits is in fact more related to a decrease in fruit softening and thus, delay of fruit decay, rather than a preservation of the total metabolic content of the fruit, which is dramatically modified by the cold. The plasticity of metabolism in peach fruits makes it possible to induce a diverse array of metabolites after cold treatment, which is successful, in some genotypes, to avoid the appearance of CI symptoms. Among these metabolites, raffinose level after long cold treatments emerges as a biomarker of mealiness resistance in peach fruit and as such could thus be a useful tool in future breeding strategies

## Author contributions

Conception and design of the work: CAB, ML, MD; Acquisition of data for the work: CAB, LM, JG, FS, GV, COB; Analysis of data for the work: CAB, LM, FS, ML, MD; Interpretation of data for the work: CAB, AF, MD; Manuscript revision and approval: CAB, LM, JG, FS, GV, COB, ML, AF, MD; Accountability: CAB, LM, JG, FS, GV, COB, ML, AF, MD.

## Funding

MD, ML, and CAB are members of the Researcher Career of CONICET and LM is a fellow of the same institution. Financial support was provided by Consejo Nacional de Investigaciones Científicas y Técnicas (CONICET, Argentina) and Agencia Nacional de Promoción de Actividades Científicas y Técnicas (Argentina, PICT 2012-416). Funding has been provided by the Alexander von Humboldt Foundation to CAB, who was recipient of a Georg Forster fellowship at AF laboratory.

### Conflict of interest statement

The authors declare that the research was conducted in the absence of any commercial or financial relationships that could be construed as a potential conflict of interest.

## Abbreviations

CI: chilling injury
CS: cold-stored fruits
EL: Elegant Lady
FD: Flordaking
H: harvested fruits
LM: Limón Marelli
R2: Rojo 2
RG: Red Globe
SL: Springlady
RS: room temperature-stored fruits.
