# Supplementary material for: Differential Metabolic Rearrangements after Cold Storage Are Correlated with Chilling Injury Resistance of Peach Fruits
Source: Front Plant Sci. 2016 Sep 30;7:1478. doi: 10.3389/fpls.2016.01478 (PMC5044465; doi:10.3389/fpls.2016.01478)
Supplement: Supplementary file 1 [file Table1.PDF]

Supplemental Table 1. Firmness and soluble solids of fruits from the six peach varieties selected in the present work. Firmness and soluble solids were measured in peach fruits at harvest (H); after ripeness at 20 °C (RS); and after cold storage at 0 °C for short time (CS) or 21 days (CS21), after which, fruits were stored at 20 °C for ripeness (CS21+RS). Different letters within each parameter indicate statistically significant differences ($p<0.05$)

Cultivar	Parameter	H	RS	CS	CS21	CS21+RS
**Flordaking (FD)**	Firmness (N)	53.9 ± 10.8 gh	10.8 ± 2.9 b	59.4 ± 3.7 hi	62.7 ± 10.8 ij	15.7 ± 8.8 b
	Soluble solids (ºBrix)	8.8 ± 0.9 abc	9.9 ± 1.5 cd	10.1 ± 0.7 de	8.7 ± 1.1 abc	9.0 ± 1.1 bc
**Rojo 2 (R2)**	Firmness (N)	50.0 ± 3.9 fg	10.8 ± 4.9 ab	42.0 ± 4.8 e	55.9 ± 5.9 h	26.5 ± 10.8 d
	Soluble solids (ºBrix)	8.1 ± 0.8 a	9.1 ± 1.0 bc	8.9 ± 1.0 abc	8.8 ± 1.0 abc	8.3 ± 1.0 ab
**Springlady (SL)**	Firmness (N)	45.1 ± 3.9 ef	8.8 ± 4.9 ab	64.9 ± 9.5 j	41.2 ± 4.9 e	4.9 ± 0.0 a
	Soluble solids (ºBrix)	11.2 ± 0.9 fgh	11.5 ± 0.9 gh	11.1 ± 1.0 fg	10.3 ± 0.8 e	11.3 ± 0.9 fgh
**Red Globe (RG)**	Firmness (N)	72.5 ± 9.8 k	27.4 ± 14.7 c	84.8 ± 13.1 l	70.6 ± 5.9 k	13.7 ± 10.8 ab
	Soluble solids (ºBrix)	12.8 ± 0.9 jkl	12.8 ± 0.9 jkl	13.4 ± 0.4 klmn	13.4 ± 1.3 lmn	13.2 ± 0.9 klm
**Elegant Lady (EL)**	Firmness (N)	64.7 ± 4.9 ij	10.8 ± 4.9 b	71.3 ± 4.3 k	58.8 ± 15.7 ij	6.9 ± 1.0 ab
	Soluble solids (ºBrix)	12.9 ± 0.8 jkl	12.6 ± 1.0 ijk	13.1 ± 0.7 kl	12.8 ± 1.4 jkl	13.1 ± 0.7 klm
**Limón Marelli (LM)**	Firmness (N)	41.7 ± 6.4 e	20.8 ± 4.5 c	47.3 ± 9.6 f	45.3 ± 7.3 ef	7.7 ± 1.6 ab
	Soluble solids (ºBrix)	12.3 ± 0.8 ij	12.0 ± 1.0 hi	10.6 ± 0.7 ef	13.9 ± 0.7 mn	14.1 ± 1.5 n
