# Supplementary material for: Differential Metabolic Rearrangements after Cold Storage Are Correlated with Chilling Injury Resistance of Peach Fruits
Source: Front Plant Sci. 2016 Sep 30;7:1478. doi: 10.3389/fpls.2016.01478 (PMC5044465; doi:10.3389/fpls.2016.01478)
Supplement: Supplementary file 2 [file Table2.PDF]

Supplemental Table 2. Relative levels of 51 metabolites analyzed by GC-MS during ripening and after cold storage of fruits from six different varieties. Values represent the mean of 5 independent determinations with three technical repetitions each one, relative to the amount found in harvested Elegant Lady (EL) fruits. Standard errors (SE) are shown. nd: not detected

	Elegant Lady (EL)									
	**H**	SE	**RS**	SE	**CS**	SE	**CS21**	SE	**CS21+RS**	SE
**Sugars**										
Fucose	1.00	0.06	2.58	0.11	1.03	0.10	1.11	0.09	2.46	0.09
Fructose	1.00	0.04	1.11	0.05	1.18	0.12	2.22	0.46	2.78	0.65
Fructose-6-P	1.00	0.13	1.15	0.29	1.06	0.06	0.99	0.10	1.54	0.23
Glucose	1.00	0.10	0.97	0.12	1.15	0.28	2.53	0.69	3.55	1.06
Glucoheptose	1.00	0.10	0.83	0.06	0.97	0.16	0.91	0.09	1.04	0.11
Isomaltose	1.00	0.16	0.48	0.06	0.97	0.20	0.97	0.17	0.93	0.04
1-O-Methyl-mannoside	1.00	0.05	1.22	0.21	0.87	0.08	0.85	0.03	0.74	0.04
Maltose	1.00	0.05	0.90	0.03	1.09	0.13	0.95	0.12	1.19	0.17
Raffinose	1.00	0.09	0.77	0.15	1.04	0.05	17.80	2.99	1.25	0.18
Rhamnose	1.00	0.06	1.03	0.05	0.99	0.24	0.89	0.09	0.97	0.12
Sucrose	1.00	0.09	1.11	0.10	1.20	0.25	3.00	0.61	3.68	0.71
Trehalose	1.00	0.07	0.42	0.06	0.57	0.06	0.45	0.04	0.56	0.07
Xylose	1.00	0.10	1.32	0.07	1.02	0.07	1.05	0.07	2.15	0.15
**Sugars alcohol**										
Galactinol	1.00	0.13	1.01	0.16	36.38	7.02	30.34	4.85	0.73	0.08
Glycerol	1.00	0.04	1.03	0.04	0.93	0.05	1.00	0.06	0.81	0.03
Myo-inositol	1.00	0.08	1.28	0.09	1.39	0.38	1.93	0.42	1.56	0.18
Maltitol	1.00	0.10	0.68	0.07	0.94	0.14	0.81	0.12	1.01	0.15
Sorbitol	1.00	0.06	1.06	0.04	1.27	0.30	2.49	0.54	2.87	0.70
**Organic acids**										
Benzoate	1.00	0.08	1.98	0.38	1.53	0.40	1.27	0.06	1.06	0.08
Citrate	1.00	0.04	1.04	0.03	1.12	0.07	2.33	0.31	2.88	0.48
Dehydroascorbate	1.00	0.19	0.74	0.19	0.74	0.14	0.41	0.11	0.60	0.09
2-oxo-Glutarate	1.00	0.12	nd		0.30	0.04	nd		nd	
Fumarate	1.00	0.18	2.44	0.48	1.63	0.28	0.97	0.05	0.68	0.08
Glycerate	1.00	0.03	2.05	0.38	1.14	0.22	0.94	0.09	1.64	0.17
Gulonate-1,4-lactone	1.00	0.05	0.67	0.17	0.85	0.12	0.74	0.16	0.70	0.09
Quinate	1.00	0.05	1.06	0.07	1.09	0.08	1.88	0.42	2.36	0.51
Malate	1.00	0.04	0.92	0.04	1.02	0.07	1.70	0.29	1.83	0.38
Succinate	1.00	0.07	0.97	0.17	0.53	0.07	nd		0.83	0.16
**Amino acids**										
Alanine	1.00	0.09	1.79	0.21	0.91	0.11	2.04	0.21	2.29	0.21
β-Alanine	1.00	0.00	nd		0.32	0.00	0.44	0.12	0.30	0.00
Asparagine	1.00	0.22	0.63	0.04	0.66	0.19	0.88	0.28	0.81	0.07
Aspartate	1.00	0.07	1.14	0.14	1.67	0.13	2.60	0.02	1.27	0.07
GABA	1.00	0.20	0.81	0.06	1.09	0.08	1.94	0.22	0.76	0.03
Glutamate	1.00	0.08	0.65	0.11	1.20	0.10	1.05	0.15	0.77	0.04
Glycine	1.00	0.25	1.02	0.21	1.18	0.20	1.27	0.20	0.86	0.11
Isoleucine	1.00	0.17	2.04	0.08	2.00	0.30	4.53	0.31	2.11	0.60
Phenylalanine	1.00	0.12	0.86	0.26	1.70	0.31	3.75	0.46	3.07	1.14
Proline	1.00	0.22	0.33	0.09	0.75	0.16	0.76	0.15	0.72	0.16
4-OH-Proline	1.00	0.14	0.42	0.18	0.60	0.09	0.65	0.15	0.38	0.06
Serine	1.00	0.08	2.10	0.11	1.45	0.12	1.87	0.17	2.98	0.19
Threonine	1.00	0.16	1.14	0.18	1.29	0.23	1.04	0.12	1.34	0.15
Valine	1.00	0.14	1.93	0.30	1.56	0.20	3.35	0.20	1.38	0.29
**Fatty acids**										
Hexadecenoic acid	1.00	0.03	0.86	0.05	0.90	0.05	0.88	0.01	0.97	0.10
Octadecanoic acid	1.00	0.03	0.68	0.10	0.87	0.08	0.91	0.04	1.06	0.16
**Miscellaneous**										
Ornithine	1.00	0.29	1.51	0.17	0.84	0.20	1.49	0.74	1.26	0.37
Phosphate	1.00	0.18	0.92	0.07	1.19	0.09	1.19	0.21	1.00	0.03
Putrescine	1.00	0.21	0.31	0.07	0.70	0.11	0.71	0.13	0.90	0.13
cis-3-caffeoylquinic acid	1.00	0.25	0.63	0.00	0.76	0.18	0.69	0.26	1.05	0.16
trans-3-caffeoylquinic acid	1.00	0.16	1.11	0.00	0.44	0.11	0.64	0.03	1.61	0.00
Spermidine	1.00	0.22	0.68	0.08	0.79	0.13	0.91	0.17	1.66	0.23
Urea	1.00	0.35	0.47	0.00	0.64	0.22	0.68	0.31	1.14	0.25

	Red Globe (RG)									
	**H**	SE	**RS**	SE	**CS**	SE	**CS21**	SE	**CS21+RS**	SE
**Sugars**										
Fucose	1.19	0.06	3.03	0.13	1.33	0.04	1.50	0.09	2.80	0.06
Fructose	0.95	0.04	0.95	0.04	0.88	0.02	0.88	0.03	0.93	0.04
Fructose-6-P	0.70	0.13	0.76	0.03	0.75	0.18	1.20	0.10	1.31	0.06
Glucose	0.85	0.06	0.77	0.07	0.72	0.09	0.66	0.06	0.72	0.02
Glucoheptose	0.66	0.09	0.60	0.10	0.66	0.07	0.72	0.11	0.60	0.23
Isomaltose	0.48	0.07	0.46	0.06	0.48	0.06	0.75	0.10	1.24	0.23
1-O-Methyl-mannoside	1.41	0.17	1.39	0.13	1.34	0.13	1.45	0.14	1.41	0.13
Maltose	0.72	0.06	0.78	0.04	0.79	0.05	0.82	0.05	0.87	0.07
Raffinose	1.04	0.16	1.15	0.10	2.00	0.22	21.65	0.88	2.34	0.33
Rhamnose	0.93	0.13	1.18	0.08	0.75	0.05	0.98	0.10	1.24	0.04
Sucrose	1.01	0.11	1.03	0.09	0.98	0.09	1.01	0.08	0.94	0.08
Trehalose	0.70	0.12	0.83	0.08	0.99	0.06	0.98	0.08	1.08	0.08
Xylose	0.92	0.08	1.17	0.05	0.92	0.04	1.00	0.07	1.94	0.19
**Sugars alcohol**										
Galactinol	0.88	0.09	1.24	0.10	44.74	6.97	76.93	15.08	1.39	0.20
Glycerol	1.10	0.06	1.09	0.06	1.04	0.05	0.96	0.03	1.00	0.04
Myo-inositol	0.95	0.09	1.12	0.07	1.10	0.09	1.05	0.04	1.08	0.10
Maltitol	0.66	0.08	0.60	0.06	0.61	0.07	0.60	0.04	0.71	0.07
Sorbitol	0.93	0.03	0.82	0.04	0.86	0.01	0.87	0.04	0.83	0.05
**Organic acids**										
Benzoate	1.73	0.14	1.60	0.17	1.44	0.15	1.55	0.15	1.54	0.12
Citrate	1.04	0.04	0.98	0.03	0.92	0.02	0.89	0.04	0.90	0.04
Dehydroascorbate	0.91	0.19	1.08	0.09	0.46	0.04	0.80	0.12	0.90	0.06
2-oxo-Glutarate	1.49	0.18	0.21	0.03	0.58	0.13	0.22	0.02	0.18	0.01
Fumarate	2.05	0.18	1.60	0.06	1.77	0.15	1.57	0.14	0.96	0.10
Glycerate	1.01	0.13	1.09	0.09	1.07	0.17	1.07	0.13	1.63	0.19
Gulonate-1,4-lactone	0.93	0.10	0.64	0.05	0.62	0.06	0.61	0.07	0.64	0.03
Quinate	0.99	0.04	1.00	0.07	0.91	0.02	0.93	0.03	0.98	0.07
Malate	0.93	0.02	0.83	0.02	0.86	0.03	0.79	0.02	0.81	0.03
Succinate	1.41	0.13	1.08	0.16	0.66	0.04	0.48	0.06	0.74	0.06
**Amino acids**										
Alanine	0.96	0.16	1.63	0.37	0.88	0.16	2.47	0.16	1.94	0.33
β-Alanine	0.52	0.10	0.56	0.03	0.56	0.02	0.48	0.02	0.56	0.04
Asparagine	1.29	0.08	1.21	0.09	1.19	0.17	1.05	0.07	1.08	0.13
Aspartate	1.32	0.06	0.78	0.05	1.68	0.24	2.15	0.19	0.81	0.10
GABA	1.05	0.06	0.86	0.05	1.01	0.13	1.83	0.10	1.19	0.09
Glutamate	0.92	0.05	0.75	0.12	1.06	0.13	1.11	0.11	0.80	0.05
Glycine	2.03	0.18	0.92	0.14	1.64	0.31	1.98	0.07	1.14	0.18
Isoleucine	4.18	0.93	3.38	1.15	4.01	0.86	6.98	0.43	5.26	1.75
Phenylalanine	2.09	0.31	2.43	0.71	2.66	0.36	5.02	1.40	8.99	3.02
Proline	2.69	0.37	0.52	0.07	1.72	0.41	1.32	0.10	1.00	0.15
4-OH-Proline	0.78	0.15	0.93	0.13	1.02	0.23	1.07	0.15	0.89	0.11
Serine	1.52	0.20	1.72	0.18	1.51	0.22	2.28	0.08	3.09	0.14
Threonine	1.65	0.23	1.20	0.23	1.59	0.33	1.63	0.11	2.26	0.43
Valine	2.80	0.58	2.14	0.55	2.52	0.55	4.62	0.32	2.70	0.68
**Fatty acids**										
Hexadecenoic acid	1.06	0.10	0.97	0.04	0.90	0.02	0.88	0.03	0.96	0.10
Octadecanoic acid	1.21	0.16	1.04	0.04	0.93	0.04	0.90	0.03	1.03	0.11
**Miscellaneous**										
Ornithine	0.60	0.08	0.58	0.12	0.71	0.25	0.83	0.13	1.12	0.33
Phosphate	1.35	0.12	1.26	0.17	1.40	0.18	1.27	0.02	1.31	0.19
Putrescine	0.75	0.08	0.69	0.12	0.75	0.22	0.79	0.07	0.90	0.19
cis-3-caffeoylquinic acid	0.63	0.18	0.58	0.10	0.38	0.07	0.61	0.13	1.18	0.22
trans-3-caffeoylquinic acid	1.71	0.30	2.13	0.47	0.98	0.27	1.05	0.12	1.69	0.23
Spermidine	1.78	0.29	1.58	0.10	1.42	0.19	1.53	0.20	2.46	0.23
Urea	0.68	0.27	0.47	0.07	0.42	0.08	0.47	0.15	0.25	0.01

	Limón Marelli (LM)									
	**H**	SE	**RS**	SE	**CS**	SE	**CS21**	SE	**CS21+RS**	SE
**Sugars**										
Fucose	0.86	0.06	1.90	0.10	0.85	0.03	0.94	0.05	1.97	0.09
Fructose	0.96	0.03	0.95	0.02	0.94	0.01	0.91	0.02	0.90	0.02
Fructose-6-P	0.80	0.12	1.08	0.07	0.79	0.06	1.03	0.11	1.46	0.13
Glucose	0.76	0.08	0.75	0.05	0.77	0.08	0.95	0.13	0.74	0.15
Glucoheptose	0.95	0.08	0.97	0.10	1.05	0.07	1.22	0.14	1.19	0.10
Isomaltose	0.93	0.11	0.91	0.11	1.01	0.23	1.52	0.17	1.42	0.19
1-O-Methyl-mannoside	1.48	0.07	1.54	0.02	1.55	0.04	1.58	0.04	1.44	0.03
Maltose	0.96	0.08	1.10	0.10	1.04	0.08	1.24	0.12	1.11	0.07
Raffinose	0.76	0.08	1.04	0.11	1.41	0.15	18.00	0.64	2.04	0.14
Rhamnose	1.00	0.06	1.07	0.10	0.91	0.07	0.96	0.07	1.30	0.11
Sucrose	1.01	0.05	1.05	0.01	0.97	0.03	1.04	0.03	0.96	0.03
Trehalose	0.86	0.09	1.05	0.09	0.98	0.13	1.28	0.07	1.14	0.09
Xylose	0.73	0.04	0.99	0.04	0.84	0.01	0.98	0.08	1.28	0.04
**Sugars alcohol**										
Galactinol	1.08	0.20	13.63	3.43	50.86	4.93	149.24	21.84	1.95	0.29
Glycerol	1.19	0.07	1.08	0.03	1.17	0.17	1.20	0.24	1.05	0.04
Myo-inositol	0.89	0.07	0.80	0.05	0.73	0.06	0.83	0.09	0.83	0.06
Maltitol	0.97	0.08	1.14	0.13	1.10	0.09	1.33	0.14	1.14	0.08
Sorbitol	0.87	0.02	0.81	0.01	0.84	0.01	0.83	0.01	0.78	0.01
**Organic acids**										
Benzoate	1.65	0.06	1.70	0.05	1.69	0.03	1.55	0.08	1.64	0.03
Citrate	0.87	0.02	0.90	0.03	0.88	0.04	0.78	0.03	0.72	0.03
Dehydroascorbate	0.66	0.06	0.79	0.18	0.39	0.06	0.57	0.09	0.35	0.02
2-oxo-Glutarate	0.50	0.02	0.20	0.00	0.32	0.04	0.20	0.02	0.14	0.00
Fumarate	2.04	0.06	2.18	0.10	2.22	0.13	2.30	0.16	1.58	0.04
Glycerate	0.73	0.03	0.91	0.06	1.27	0.07	1.05	0.05	1.33	0.14
Gulonate-1,4-lactone	0.64	0.06	0.63	0.08	0.68	0.06	0.79	0.10	0.62	0.04
Quinate	0.87	0.03	0.81	0.04	0.84	0.02	0.85	0.03	0.78	0.04
Malate	0.93	0.02	0.93	0.01	0.94	0.03	0.95	0.01	0.84	0.01
Succinate	1.11	0.11	0.50	0.03	0.58	0.02	0.57	0.05	0.61	0.07
**Amino acids**										
Alanine	1.00	0.14	5.38	0.47	1.22	0.09	2.44	0.46	7.08	0.29
β-Alanine	nd		0.45	0.02	nd		nd		0.61	0.05
Asparagine	0.34	0.07	0.21	0.04	0.48	0.05	0.54	0.10	0.22	0.04
Aspartate	0.81	0.08	1.06	0.05	1.32	0.10	1.80	0.13	0.84	0.08
GABA	0.75	0.02	1.41	0.15	1.27	0.06	2.25	0.29	1.91	0.21
Glutamate	0.97	0.09	1.46	0.03	1.27	0.09	1.37	0.07	1.34	0.05
Glycine	0.73	0.07	0.40	0.04	0.82	0.09	0.96	0.08	0.73	0.09
Isoleucine	1.45	0.16	0.52	0.05	2.37	0.27	5.18	0.41	0.61	0.06
Phenylalanine	0.85	0.06	0.44	0.05	1.62	0.23	3.78	0.37	0.89	0.31
Proline	0.44	0.07	0.48	0.07	0.72	0.12	0.87	0.04	0.77	0.06
4-OH-Proline	0.41	0.05	0.44	0.05	0.26	0.05	0.44	0.05	0.54	0.15
Serine	1.00	0.11	2.27	0.15	1.31	0.10	1.78	0.16	2.86	0.15
Threonine	0.61	0.09	0.52	0.05	0.82	0.10	0.95	0.08	0.85	0.06
Valine	1.25	0.08	0.91	0.08	1.71	0.18	3.42	0.26	1.18	0.13
**Fatty acids**										
Hexadecenoic acid	0.93	0.02	0.92	0.03	0.87	0.03	0.79	0.03	0.81	0.02
Octadecanoic acid	0.94	0.02	0.95	0.06	0.85	0.04	0.74	0.05	0.78	0.04
**Miscellaneous**										
Ornithine	0.42	0.09	0.35	0.07	1.20	0.20	1.11	0.18	0.92	0.40
Phosphate	0.85	0.05	0.79	0.03	0.96	0.08	1.13	0.05	1.11	0.10
Putrescine	0.32	0.07	0.23	0.05	0.74	0.13	0.65	0.08	0.50	0.19
cis-3-caffeoylquinic acid	1.67	0.35	1.48	0.34	0.38	0.09	1.60	0.49	0.30	0.06
trans-3-caffeoylquinic acid	1.86	0.45	1.23	0.28	0.33	0.06	1.62	0.40	0.90	0.43
Spermidine	nd		nd		nd		0.41	0.00	0.34	0.00
Urea	0.47	0.07	0.37	0.12	0.47	0.14	1.61	1.30	0.29	0.02

	Springlady (SL)									
	**H**	SE	**RS**	SE	**CS**	SE	**CS21**	SE	**CS21+RS**	SE
**Sugars**										
Fucose	0.83	0.11	2.36	0.10	1.12	0.07	0.99	0.07	2.64	0.14
Fructose	0.96	0.03	0.96	0.03	0.98	0.03	0.95	0.03	0.93	0.01
Fructose-6-P	0.34	0.05	0.39	0.01	0.40	0.04	0.32	0.02	0.43	0.04
Glucose	0.61	0.10	0.63	0.05	0.52	0.04	0.52	0.02	0.57	0.02
Glucoheptose	0.25	0.06	0.32	0.06	0.37	0.07	0.20	0.03	0.48	0.05
Isomaltose	0.25	0.06	0.24	0.02	0.34	0.12	0.24	0.05	0.34	0.04
1-O-Methyl-mannoside	1.11	0.07	1.28	0.06	1.16	0.11	1.14	0.06	1.23	0.05
Maltose	0.41	0.03	0.46	0.03	0.47	0.06	0.40	0.03	0.44	0.02
Raffinose	1.21	0.09	1.64	0.08	2.05	0.14	13.71	0.94	2.29	0.08
Rhamnose	0.71	0.06	0.82	0.03	0.74	0.06	0.65	0.05	0.94	0.04
Sucrose	1.09	0.08	1.19	0.05	1.10	0.07	1.11	0.04	1.07	0.04
Trehalose	0.46	0.05	0.67	0.04	0.59	0.07	0.54	0.04	0.66	0.04
Xylose	0.61	0.03	0.99	0.05	0.74	0.05	0.82	0.03	1.54	0.05
**Sugars alcohol**										
Galactinol	nd		1.44	0.06	12.16	3.24	11.75	1.59	0.60	0.07
Glycerol	2.92	0.14	2.44	0.13	2.54	0.10	2.68	0.12	2.72	0.07
Myo-inositol	0.82	0.06	0.80	0.05	0.80	0.09	0.66	0.04	0.68	0.08
Maltitol	0.29	0.02	0.30	0.02	0.30	0.03	0.26	0.02	0.31	0.01
Sorbitol	1.06	0.06	0.74	0.03	0.94	0.11	0.97	0.05	0.76	0.04
**Organic acids**										
Benzoate	9.44	2.86	11.07	1.02	8.45	2.96	7.91	2.58	9.22	0.48
Citrate	1.05	0.05	0.80	0.09	1.01	0.08	0.93	0.08	0.82	0.04
Dehydroascorbate	0.70	0.09	0.50	0.05	0.75	0.18	0.38	0.06	0.56	0.07
2-oxo-Glutarate	1.21	0.14	0.38	0.03	0.49	0.05	0.17	0.00	0.42	0.06
Fumarate	0.87	0.04	0.77	0.10	0.80	0.05	0.64	0.07	0.37	0.02
Glycerate	0.68	0.08	0.64	0.05	0.70	0.03	0.90	0.07	1.00	0.08
Gulonate-1,4-lactone	0.53	0.04	0.31	0.01	0.32	0.04	0.35	0.05	0.28	0.04
Quinate	1.07	0.08	1.09	0.03	1.13	0.05	1.06	0.03	1.08	0.04
Malate	1.16	0.03	1.03	0.04	1.07	0.03	1.04	0.02	0.95	0.02
Succinate	3.31	0.38	1.48	0.08	1.25	0.15	0.70	0.04	1.57	0.10
**Amino acids**										
Alanine	2.88	0.68	1.12	0.26	3.44	0.81	3.49	0.47	1.47	0.31
β-Alanine	1.59	0.15	0.49	0.04	1.32	0.38	1.13	0.26	0.88	0.14
Asparagine	1.81	0.14	1.39	0.12	1.36	0.21	1.33	0.14	1.32	0.06
Aspartate	1.00	0.12	0.70	0.06	1.32	0.18	1.83	0.29	0.81	0.03
GABA	1.38	0.20	0.26	0.07	1.26	0.52	2.02	0.69	0.54	0.17
Glutamate	1.23	0.16	0.30	0.07	0.83	0.33	0.67	0.21	0.58	0.20
Glycine	5.88	2.30	0.90	0.22	2.99	0.56	3.35	0.75	1.77	0.14
Isoleucine	7.16	1.71	6.83	1.52	10.25	1.85	11.46	2.10	14.98	1.91
Phenylalanine	4.78	0.79	5.40	1.37	7.41	0.79	11.28	2.60	11.53	1.35
Proline	3.24	0.49	0.84	0.20	2.71	0.65	2.32	0.61	1.49	0.15
4-OH-Proline	0.46	0.08	0.24	0.04	0.34	0.07	0.42	0.10	0.29	0.04
Serine	2.65	0.41	2.96	0.35	3.73	0.55	3.38	0.71	6.39	0.70
Threonine	2.84	0.42	3.01	0.48	3.89	0.59	2.82	0.53	5.33	0.38
Valine	5.53	1.25	3.70	0.46	7.64	1.52	8.42	1.73	7.06	1.30
**Fatty acids**										
Hexadecenoic acid	0.88	0.03	0.81	0.05	0.80	0.03	0.95	0.05	0.80	0.02
Octadecanoic acid	0.69	0.03	0.67	0.05	0.76	0.04	0.81	0.07	0.67	0.04
**Miscellaneous**										
Ornithine	0.19	0.00	1.41	0.26	1.74	0.65	1.41	0.02	1.94	0.55
Phosphate	1.03	0.06	1.36	0.17	1.41	0.07	1.08	0.07	1.30	0.08
Putrescine	0.25	0.04	0.20	0.07	0.71	0.16	0.25	0.06	0.64	0.06
cis-3-caffeoylquinic acid	0.89	0.25	0.91	0.21	0.88	0.21	0.97	0.13	0.89	0.12
trans-3-caffeoylquinic acid	1.35	0.44	1.43	0.38	1.63	0.43	1.41	0.22	1.67	0.14
Spermidine	0.91	0.00	0.44	0.13	0.44	0.00	nd		0.61	0.03
Urea	1.01	0.31	0.73	0.14	1.06	0.17	0.70	0.10	0.89	0.35

	Rojo 2 (R2)									
	**H**	SE	**RS**	SE	**CS**	SE	**CS21**	SE	**CS21+RS**	SE
**Sugars**										
Fucose	0.51	0.03	1.61	0.12	0.62	0.02	0.62	0.01	1.17	0.09
Fructose	0.91	0.01	0.92	0.02	0.91	0.03	0.91	0.03	0.83	0.01
Fructose-6-P	0.37	0.00	0.34	0.03	0.32	0.03	0.32	0.02	0.31	0.01
Glucose	0.63	0.02	0.61	0.05	0.62	0.08	0.53	0.04	0.50	0.03
Glucoheptose	0.52	0.03	0.55	0.02	0.55	0.05	0.61	0.03	0.59	0.03
Isomaltose	0.11	0.01	0.12	0.01	0.16	0.03	0.13	0.01	0.15	0.02
1-O-Methyl-mannoside	0.82	0.07	0.90	0.06	0.90	0.05	0.86	0.07	0.77	0.08
Maltose	0.33	0.01	0.36	0.02	0.39	0.04	0.36	0.02	0.33	0.02
Raffinose	1.17	0.08	1.45	0.10	1.56	0.12	6.69	1.01	2.09	0.23
Rhamnose	0.62	0.06	0.89	0.05	0.78	0.05	0.67	0.04	1.02	0.11
Sucrose	0.98	0.08	0.95	0.04	0.97	0.07	0.85	0.07	0.75	0.06
Trehalose	0.41	0.04	0.53	0.03	0.52	0.06	0.46	0.03	0.49	0.03
Xylose	0.63	0.04	0.99	0.11	0.75	0.03	0.99	0.03	1.79	0.20
**Sugars alcohol**										
Galactinol	nd		nd		6.89	1.43	2.40	0.36	nd	
Glycerol	1.00	0.05	1.02	0.03	1.01	0.05	0.97	0.05	0.94	0.05
Myo-inositol	0.84	0.08	0.83	0.08	0.97	0.09	0.69	0.04	0.60	0.07
Maltitol	0.22	0.01	0.24	0.01	0.27	0.03	0.25	0.02	0.21	0.01
Sorbitol	0.68	0.12	0.33	0.04	0.70	0.12	0.68	0.13	0.30	0.04
**Organic acids**										
Benzoate	1.13	0.03	1.01	0.05	1.11	0.05	1.15	0.04	0.98	0.05
Citrate	1.06	0.02	0.99	0.04	1.02	0.02	1.02	0.04	0.81	0.04
Dehydroascorbate	0.31	0.02	0.35	0.02	0.30	0.04	0.34	0.04	0.31	0.04
2-oxo-Glutarate	0.57	0.07	0.34	0.02	0.45	0.04	nd		0.35	0.07
Fumarate	0.83	0.05	0.96	0.07	0.93	0.05	0.72	0.06	0.52	0.05
Glycerate	1.20	0.08	1.54	0.19	1.29	0.06	1.12	0.06	1.39	0.07
Gulonate-1,4-lactone	0.57	0.04	0.43	0.03	0.48	0.06	0.45	0.02	0.40	0.02
Quinate	0.94	0.03	0.94	0.01	0.99	0.04	0.96	0.05	0.83	0.02
Malate	0.86	0.04	0.88	0.05	0.91	0.03	0.86	0.04	0.79	0.03
Succinate	5.51	1.15	3.85	0.58	2.83	0.39	0.78	0.16	2.18	0.37
**Amino acids**										
Alanine	4.48	0.98	4.31	0.84	3.38	0.64	5.75	0.75	9.70	1.12
β-Alanine	1.41	0.10	1.12	0.14	1.15	0.15	1.40	0.16	1.10	0.22
Asparagine	1.30	0.13	1.24	0.07	1.23	0.17	1.26	0.09	1.17	0.06
Aspartate	1.50	0.19	1.20	0.07	2.03	0.12	3.36	0.30	1.41	0.09
GABA	1.95	0.22	1.53	0.20	1.71	0.18	3.47	0.37	1.87	0.31
Glutamate	1.04	0.11	0.77	0.04	1.36	0.18	0.48	0.06	0.74	0.06
Glycine	2.99	0.62	2.07	0.21	2.15	0.20	2.77	0.31	2.69	0.36
Isoleucine	12.99	3.51	16.95	2.14	11.62	2.07	17.34	1.61	21.12	2.79
Phenylalanine	5.13	1.58	6.68	1.23	4.59	0.77	9.99	1.40	10.28	2.25
Proline	6.59	1.35	3.09	0.77	4.18	0.71	5.07	0.81	3.74	1.06
4-OH-Proline	0.79	0.10	0.84	0.11	0.84	0.19	0.76	0.14	0.55	0.13
Serine	5.18	1.17	5.66	0.80	4.18	0.56	5.47	0.53	7.96	0.71
Threonine	4.06	0.86	3.97	0.52	3.14	0.47	4.21	0.48	4.84	0.49
Valine	9.66	2.48	10.74	1.24	8.42	1.52	11.55	0.98	13.40	1.60
**Fatty acids**										
Hexadecenoic acid	1.00	0.02	1.00	0.03	0.98	0.02	1.07	0.06	0.92	0.02
Octadecanoic acid	1.12	0.05	1.08	0.04	1.06	0.04	1.26	0.11	1.04	0.01
**Miscellaneous**										
Ornithine	0.67	0.20	0.81	0.09	0.87	0.26	1.24	0.17	1.26	0.15
Phosphate	1.09	0.04	1.14	0.08	1.27	0.08	1.22	0.08	1.18	0.06
Putrescine	1.01	0.22	0.95	0.10	1.10	0.22	1.32	0.13	1.25	0.07
cis-3-caffeoylquinic acid	0.32	0.03	0.30	0.02	0.36	0.09	0.30	0.02	0.26	0.03
trans-3-caffeoylquinic acid	0.45	0.05	0.40	0.03	0.50	0.14	0.44	0.07	0.36	0.09
Spermidine	0.43	0.04	0.55	0.03	0.40	0.07	0.44	0.04	0.65	0.04
Urea	0.66	0.07	0.56	0.08	1.12	0.39	0.44	0.06	0.47	0.07

	Flordaking (FD)									
	**H**	SE	**RS**	SE	**CS**	SE	**CS21**	SE	**CS21+RS**	SE
**Sugars**										
Fucose	0.86	0.11	2.00	0.07	0.94	0.03	1.18	0.25	2.79	0.12
Fructose	1.04	0.02	1.06	0.03	1.05	0.01	1.10	0.04	1.03	0.02
Fructose-6-P	0.41	0.00	0.34	0.02	0.31	0.02	0.44	0.07	0.46	0.04
Glucose	0.84	0.02	0.93	0.03	0.92	0.08	0.73	0.06	0.93	0.07
Glucoheptose	0.80	0.08	0.91	0.05	0.97	0.06	0.61	0.03	1.27	0.04
Isomaltose	0.14	0.02	0.16	0.01	0.26	0.02	0.21	0.04	0.28	0.02
1-O-Methyl-mannoside	0.72	0.07	0.75	0.02	0.82	0.09	0.65	0.07	0.72	0.02
Maltose	0.40	0.05	0.42	0.01	0.49	0.03	0.40	0.03	0.44	0.02
Raffinose	1.79	0.24	2.13	0.14	2.70	0.20	5.65	1.66	3.03	0.17
Rhamnose	0.57	0.01	0.87	0.06	0.79	0.08	0.64	0.03	0.78	0.02
Sucrose	0.75	0.10	0.82	0.02	0.90	0.07	0.63	0.10	0.71	0.02
Trehalose	0.49	0.05	0.55	0.03	0.69	0.08	0.47	0.05	0.61	0.04
Xylose	0.82	0.03	1.14	0.06	0.82	0.03	1.48	0.32	2.39	0.10
**Sugars alcohol**										
Galactinol	nd		nd		13.56	2.26	57.31	24.34	nd	
Glycerol	2.66	0.29	2.42	0.12	2.50	0.09	2.70	0.27	2.54	0.11
Myo-inositol	0.68	0.04	0.66	0.03	0.79	0.02	0.66	0.06	0.67	0.02
Maltitol	0.30	0.03	0.32	0.02	0.40	0.02	0.34	0.02	0.36	0.01
Sorbitol	0.81	0.07	0.48	0.06	1.02	0.02	0.67	0.23	0.48	0.02
**Organic acids**										
Benzoate	7.45	1.01	10.40	0.60	7.89	0.55	6.52	2.56	8.92	0.41
Citrate	1.05	0.02	1.06	0.04	1.07	0.02	1.11	0.05	0.96	0.03
Dehydroascorbate	0.35	0.05	0.40	0.04	0.50	0.06	0.27	0.01	0.38	0.01
2-oxo-Glutarate	0.57	0.07	0.31	0.05	0.40	0.04	0.42	0.22	0.19	0.01
Fumarate	0.74	0.04	0.57	0.04	0.63	0.08	0.54	0.06	0.25	0.01
Glycerate	1.21	0.13	1.57	0.15	1.59	0.14	1.80	0.30	1.88	0.18
Gulonate-1,4-lactone	0.45	0.04	0.35	0.02	0.41	0.03	0.54	0.09	0.53	0.06
Quinate	1.22	0.05	1.18	0.05	1.25	0.03	1.32	0.07	1.15	0.04
Malate	0.89	0.02	0.78	0.06	0.90	0.03	0.74	0.06	0.65	0.04
Succinate	5.46	0.71	1.87	0.35	1.27	0.06	1.64	0.55	1.72	0.27
**Amino acids**										
Alanine	1.50	0.56	0.58	0.18	0.29	0.06	4.74	1.08	2.00	0.40
β-Alanine	0.58	0.08	0.41	0.03	0.50	0.04	1.11	0.18	0.52	0.08
Asparagine	1.18	0.22	0.80	0.04	0.64	0.13	1.28	0.19	0.63	0.08
Aspartate	1.09	0.08	0.90	0.07	1.29	0.13	1.76	0.64	0.76	0.05
GABA	0.65	0.19	0.13	0.03	0.25	0.04	2.18	0.85	0.48	0.09
Glutamate	0.67	0.19	0.11	0.04	0.13	0.03	0.83	0.27	0.34	0.08
Glycine	5.29	1.74	2.73	0.44	1.90	0.28	6.15	0.76	1.23	0.13
Isoleucine	8.72	1.43	10.00	0.97	5.03	0.29	17.10	4.19	16.77	2.19
Phenylalanine	5.62	0.83	6.25	0.85	3.84	0.85	10.91	2.25	11.84	1.73
Proline	1.28	0.11	1.12	0.47	2.08	0.38	4.07	1.05	1.06	0.26
4-OH-Proline	0.48	0.04	0.29	0.05	0.46	0.07	0.58	0.13	0.35	0.05
Serine	2.61	0.29	3.31	0.63	1.92	0.37	4.84	1.08	6.57	0.63
Threonine	3.05	0.37	2.99	0.47	2.66	0.38	3.59	0.62	4.59	0.76
Valine	4.60	1.18	3.84	0.41	2.18	0.19	9.87	2.81	7.15	0.71
**Fatty acids**										
Hexadecenoic acid	0.80	0.04	0.75	0.03	0.64	0.02	0.90	0.05	0.72	0.03
Octadecanoic acid	0.65	0.04	0.65	0.04	0.58	0.03	0.70	0.04	0.58	0.03
**Miscellaneous**										
Ornithine	1.85	0.94	4.39	0.38	7.53	0.84	1.32	1.03	3.29	0.99
Phosphate	1.04	0.10	1.50	0.22	1.43	0.18	1.20	0.08	1.23	0.18
Putrescine	0.80	0.18	0.53	0.06	0.88	0.16	0.76	0.10	0.79	0.12
cis-3-caffeoylquinic acid	1.83	0.53	4.17	1.30	0.51	0.23	0.92	0.20	4.06	0.56
trans-3-caffeoylquinic acid	3.87	1.61	6.09	1.82	0.65	0.36	0.95	0.21	4.40	0.64
Spermidine	0.47	0.06	0.70	0.14	nd		0.81	0.16	1.39	0.11
Urea	1.10	0.34	1.23	0.11	0.92	0.06	1.48	0.39	0.86	0.16
