# Supplementary material for: Differential Metabolic Rearrangements after Cold Storage Are Correlated with Chilling Injury Resistance of Peach Fruits
Source: Front Plant Sci. 2016 Sep 30;7:1478. doi: 10.3389/fpls.2016.01478 (PMC5044465; doi:10.3389/fpls.2016.01478)
Supplement: Supplementary file 3 [file Table3.PDF]

Supplemental Table 3. Variable contribution to the principal components (%) shown in Figure 4. Values higher than 4% are highlighted in yellow.

Variable	PC1	PC2	PC3
Fucose	0.291	3.484	0.132
Fructose	0.647	1.967	9.615
Fructose-6-P	5.217	0.007	0.096
Glucose	1.829	2.295	6.355
Glucoheptose	1.971	0.613	0.016
Isomaltose	5.096	0.106	0.037
1-O-Methyl-mannoside	1.965	0.466	4.423
Maltose	5.896	0.082	0.000
Raffinose	0.147	0.024	1.175
Rhamnose	3.018	0.009	0.575
Sucrose	1.482	0.365	6.852
Trehalose	2.216	0.005	3.815
Xylose	0.029	1.513	0.707
Galactinol	0.485	0.370	0.051
Glycerol	2.317	4.423	0.802
Myo-inositol	2.727	0.008	4.644
Maltitol	5.692	0.120	0.046
Sorbitol	2.035	0.650	4.496
Benzoate	1.775	5.986	0.844
Citrate	0.227	0.644	11.429
Dehydroascorbate	2.004	0.181	0.442
2-oxo-Glutarate	0.524	0.146	4.691
Fumarate	3.542	1.713	1.020
Glycerate	0.039	0.285	0.632
Gulonate-1,4-lactone	3.620	0.837	0.326
Quinate	0.005	3.462	9.406

Variable	PC1	PC2	PC3
Malate	0.850	0.242	6.155
Succinate	2.659	0.023	1.194
Alanine	0.237	4.129	0.171
β-Alanine	2.290	0.713	0.679
Asparagine	2.608	0.411	1.571
Aspartate	0.044	3.529	4.051
GABA	0.065	8.775	0.649
Glutamate	1.330	5.523	0.017
Glycine	4.063	0.242	0.531
Isoleucine	4.973	0.066	0.579
Phenylalanine	4.246	0.041	0.982
Proline	3.742	3.007	0.433
4-OH-Proline	0.079	6.019	0.551
Serine	3.579	0.062	0.351
Threonine	5.582	0.003	0.287
Valine	4.842	0.913	0.448
Hexadecenoic acid	0.170	7.162	0.854
Octadecanoic acid	0.353	7.519	0.993
Ornithine	0.758	4.766	0.341
Phosphate	1.439	0.279	0.171
Putrescine	0.348	1.421	2.247
cis-3-caffeoylquinic acid	0.007	7.039	0.245
trans-3-caffeoylquinic acid	0.012	6.252	0.592
Spermidine	0.040	0.012	2.152
Urea	0.894	2.088	1.127
