# Supplementary material for: Differential Metabolic Rearrangements after Cold Storage Are Correlated with Chilling Injury Resistance of Peach Fruits
Source: Front Plant Sci. 2016 Sep 30;7:1478. doi: 10.3389/fpls.2016.01478 (PMC5044465; doi:10.3389/fpls.2016.01478)
Supplement: Supplementary file 4 [file Table4.PDF]

Supplemental Table 4. Metabolite fold change between CS and H, CS21 and H, and CS21+RS and H. Metabolites that showed statistically significant changes ($p < 0.05$) are indicated in blue (increase, ratios higher than 1) or red (decrease, ratios lower than 1). ↑ or ↓ means increase or decrease,respectivly, from (or to) non detectable levels. Nc: no change

	Elegant Lady (EL) (Resistant)			*Red Globe (RG) (Resistant)*			*Limón Marelli (LM) (Resistant)*		
	**CS/H**	**CS21/H**	**CS21+RS/H**	**CS/H**	**CS21/H**	**CS21+RS/H**	**CS/H**	**CS21/H**	**CS21+RS/H**
**Sugars**									
Fucose	1.03	1.11	2.46	1.12	1.26	2.36	0.99	1.09	2.30
Fructose	1.18	2.22	2.78	0.93	0.93	0.98	0.98	0.96	0.94
Fructose-6-P	1.06	0.99	1.54	1.08	1.72	1.88	0.99	1.29	1.81
Glucose	1.15	2.53	3.55	0.85	0.78	0.85	1.01	1.25	0.97
Glucoheptose	0.97	0.91	1.04	1.01	1.09	0.91	1.11	1.28	1.25
Isomaltose	0.97	0.97	0.93	1.00	1.58	2.61	1.08	1.63	1.52
1-O-Methyl-mannoside	0.87	0.85	0.74	0.95	1.03	1.00	1.05	1.07	0.97
Maltose	1.09	0.95	1.19	1.09	1.14	1.20	1.08	1.29	1.16
Raffinose	1.04	17.80	1.25	1.92	20.83	2.25	1.87	23.80	2.69
Rhamnose	0.99	0.89	0.97	0.81	1.06	1.33	0.91	0.97	1.31
Sucrose	1.20	3.00	3.68	0.96	1.00	0.93	0.96	1.02	0.95
Trehalose	0.57	0.45	0.56	1.41	1.39	1.53	1.13	1.48	1.32
Xylose	1.02	1.05	2.15	0.99	1.09	2.10	1.15	1.35	1.76
**Sugars alcohol**									
Galactinol	36.38	30.34	0.73	50.57	86.95	1.57	47.23	138.59	1.81
Glycerol	0.93	1.00	0.81	0.95	0.87	0.91	0.99	1.01	0.88
Myo-inositol	1.39	1.93	1.56	1.16	1.10	1.13	0.82	0.94	0.94
Maltitol	0.94	0.81	1.01	0.92	0.90	1.07	1.13	1.36	1.17
Sorbitol	1.27	2.49	2.87	0.92	0.93	0.89	0.97	0.95	0.89
**Organic acids**									
Benzoate	1.53	1.27	1.06	0.83	0.90	0.89	1.03	0.94	0.99
Citrate	1.12	2.33	2.88	0.88	0.86	0.86	1.01	0.89	0.83
Dehydroascorbate	0.74	0.41	0.60	0.50	0.88	0.99	0.59	0.87	0.53
2-oxo-Glutarate	0.30	↓	↓	0.39	0.14	0.12	0.65	0.40	0.29
Fumarate	1.63	0.97	0.68	0.86	0.77	0.47	1.09	1.13	0.77
Glycerate	1.14	0.94	1.64	1.06	1.06	1.62	1.73	1.43	1.82
Gulonate-1,4-lactone	0.85	0.74	0.70	0.66	0.66	0.69	1.06	1.23	0.97
Quinate	1.09	1.88	2.36	0.91	0.94	0.98	0.97	0.98	0.90
Malate	1.02	1.70	1.83	0.93	0.86	0.87	1.01	1.02	0.91
Succinate	0.53	↓	0.83	0.47	0.34	0.53	0.52	0.51	0.55
**Amino acids**									
Alanine	0.91	2.04	2.29	0.92	2.58	2.02	1.22	2.46	7.11
β-Alanine	0.32	0.44	0.30	1.07	0.91	1.08	nc	nc	↑
Asparagine	0.66	0.88	0.81	0.92	0.81	0.84	1.42	1.60	0.65
Aspartate	1.67	2.60	1.27	1.27	1.63	0.61	1.62	2.21	1.03
GABA	1.09	1.94	0.76	0.96	1.74	1.13	1.69	2.99	2.54
Glutamate	1.20	1.05	0.77	1.15	1.20	0.86	1.31	1.42	1.39
Glycine	1.18	1.27	0.86	0.81	0.98	0.56	1.12	1.32	1.00
Isoleucine	2.00	4.53	2.11	0.96	1.67	1.26	1.63	3.57	0.42
Phenylalanine	1.70	3.75	3.07	1.27	2.40	4.30	1.90	4.44	1.04
Proline	0.75	0.76	0.72	0.64	0.49	0.37	1.64	1.99	1.76
4-OH-Proline	0.60	0.65	0.38	1.30	1.36	1.13	0.64	1.08	1.31
Serine	1.45	1.87	2.98	0.99	1.49	2.03	1.31	1.77	2.86
Threonine	1.29	1.04	1.34	0.96	0.98	1.37	1.35	1.57	1.40
Valine	1.56	3.35	1.38	0.90	1.65	0.96	1.36	2.73	0.94
**Fatty acids**									
Hexadecenoic acid	0.90	0.88	0.97	0.84	0.83	0.91	0.94	0.86	0.88
Octadecanoic acid	0.87	0.91	1.06	0.76	0.74	0.85	0.90	0.79	0.83
**Miscellaneous**									
Ornithine	0.84	1.49	1.26	1.19	1.39	1.88	2.85	2.66	2.19
Phosphate	1.19	1.19	1.00	1.04	0.94	0.98	1.13	1.34	1.31
Putrescine	0.70	0.71	0.90	1.00	1.06	1.21	2.31	2.05	1.59
cis-3-caffeoylquinic acid	0.76	0.69	1.05	0.60	0.97	1.86	0.22	0.96	0.18
trans-3-caffeoylquinic acid	0.44	0.64	1.61	0.57	0.61	0.99	0.18	0.87	0.48
Spermidine	0.79	0.91	1.66	0.80	0.86	1.38	nc	↑	↑
Urea	0.64	0.68	1.14	0.62	0.69	0.37	0.99	3.42	0.62

	Springlady (SL) (Resistant)			Rojo 2 (R2) (Intermediate)			Flordaking (FD) (Susceptible)		
	CS/H	CS21/H	CS21+RS/H	CS/H	CS21/H	CS21+RS/H	CS/H	CS21/H	CS21+RS/H
**Sugars**									
Fucose	1.35	1.19	3.17	1.22	1.21	2.28	1.08	1.36	3.23
Fructose	1.02	0.99	0.97	1.00	1.00	0.91	1.00	1.06	0.99
Fructose-6-P	1.16	0.94	1.25	0.85	0.85	0.84	0.76	1.08	1.13
Glucose	0.84	0.85	0.93	0.98	0.84	0.79	1.09	0.87	1.11
Glucoheptose	1.47	0.81	1.91	1.06	1.17	1.14	1.22	0.76	1.60
Isomaltose	1.36	0.96	1.38	1.46	1.18	1.42	1.92	1.56	2.03
1-O-Methyl-mannoside	1.04	1.03	1.10	1.10	1.04	0.93	1.15	0.90	1.00
Maltose	1.15	0.98	1.10	1.21	1.11	1.00	1.24	1.02	1.10
Raffinose	1.70	11.32	1.89	1.34	5.73	1.79	1.51	3.15	1.69
Rhamnose	1.05	0.92	1.32	1.26	1.08	1.66	1.39	1.12	1.36
Sucrose	1.02	1.03	0.98	0.99	0.86	0.76	1.19	0.83	0.95
Trehalose	1.28	1.17	1.43	1.24	1.10	1.17	1.40	0.96	1.24
Xylose	1.21	1.33	2.50	1.19	1.58	2.85	1.01	1.81	2.93
**Sugars alcohol**									
Galactinol	↑	↑	↑	↑	↑	nc	↑	↑	nc
Glycerol	0.87	0.92	0.93	1.01	0.97	0.94	0.94	1.01	0.95
Myo-inositol	0.98	0.81	0.83	1.16	0.83	0.71	1.16	0.96	0.98
Maltitol	1.04	0.91	1.09	1.25	1.15	0.98	1.32	1.13	1.19
Sorbitol	0.89	0.91	0.71	1.03	0.99	0.45	1.25	0.83	0.59
**Organic acids**									
Benzoate	0.90	0.84	0.98	0.98	1.01	0.86	1.06	0.88	1.20
Citrate	0.97	0.89	0.79	0.96	0.96	0.76	1.02	1.05	0.92
Dehydroascorbate	1.07	0.54	0.81	0.97	1.11	0.99	1.40	0.76	1.06
2-oxo-Glutarate	0.41	0.14	0.35	0.79	↓	0.62	0.69	0.72	0.33
Fumarate	0.91	0.74	0.42	1.12	0.87	0.62	0.85	0.72	0.34
Glycerate	1.03	1.33	1.48	1.08	0.93	1.16	1.31	1.48	1.55
Gulonate-1,4-lactone	0.61	0.67	0.54	0.85	0.79	0.71	0.91	1.21	1.18
Quinate	1.05	0.99	1.01	1.06	1.03	0.88	1.02	1.08	0.94
Malate	0.93	0.90	0.82	1.06	1.00	0.91	1.01	0.84	0.74
Succinate	0.38	0.21	0.47	0.51	0.14	0.40	0.23	0.30	0.32
**Amino acids**									
Alanine	1.20	1.21	0.51	0.75	1.28	2.16	0.19	3.16	1.33
β-Alanine	0.83	0.71	0.55	0.81	1.00	0.78	0.86	1.92	0.90
Asparagine	0.75	0.73	0.73	0.95	0.97	0.90	0.55	1.09	0.53
Aspartate	1.33	1.84	0.81	1.35	2.24	0.94	1.19	1.61	0.70
GABA	0.91	1.47	0.39	0.88	1.78	0.96	0.39	3.38	0.75
Glutamate	0.68	0.54	0.47	1.31	0.46	0.72	0.19	1.23	0.51
Glycine	0.51	0.57	0.30	0.72	0.93	0.90	0.36	1.16	0.23
Isoleucine	1.43	1.60	2.09	0.89	1.33	1.63	0.58	1.96	1.92
Phenylalanine	1.55	2.36	2.41	0.89	1.95	2.00	0.68	1.94	2.11
Proline	0.84	0.71	0.46	0.63	0.77	0.57	1.63	3.19	0.83
4-OH-Proline	0.74	0.91	0.64	1.06	0.96	0.70	0.96	1.19	0.73
Serine	1.41	1.27	2.41	0.81	1.06	1.54	0.73	1.85	2.52
Threonine	1.37	0.99	1.88	0.77	1.04	1.19	0.87	1.18	1.50
Valine	1.38	1.52	1.28	0.87	1.20	1.39	0.47	2.14	1.55
**Fatty acids**									
Hexadecenoic acid	0.91	1.08	0.91	0.98	1.07	0.92	0.80	1.12	0.90
Octadecanoic acid	1.11	1.17	0.97	0.95	1.13	0.93	0.90	1.08	0.89
**Miscellaneous**									
Ornithine	9.38	7.62	10.44	1.29	1.84	1.87	4.07	0.71	1.78
Phosphate	1.36	1.04	1.27	1.17	1.12	1.09	1.38	1.15	1.18
Putrescine	2.81	0.98	2.51	1.09	1.30	1.24	1.11	0.96	0.99
cis-3-caffeoylquinic acid	0.98	1.09	1.00	1.11	0.94	0.81	0.28	0.50	2.22
trans-3-caffeoylquinic aci	1.21	1.05	1.24	1.12	1.00	0.81	0.17	0.25	1.14
Spermidine	0.48	1.02	0.67	0.95	1.02	1.53	↓	1.71	2.94
Urea	1.05	0.69	0.89	1.71	0.66	0.71	0.84	1.34	0.78
