# Supplementary material for: Differential Metabolic Rearrangements after Cold Storage Are Correlated with Chilling Injury Resistance of Peach Fruits
Source: Front Plant Sci. 2016 Sep 30;7:1478. doi: 10.3389/fpls.2016.01478 (PMC5044465; doi:10.3389/fpls.2016.01478)
Supplement: Supplementary file 5 [file Table5.PDF]

Supplemental Table 5. Metabolite-metabolite correlations in each of the six peach fruit varieties under different postharvest conditions

Out of the 1,275 pairs of metabolites analyzed, significant positive and negative correlations ($P < 0.05$) are indicated

	Positive	Negative	Total
**EL**	76	23	99
**SL**	61	36	97
**LM**	92	44	136
**FD**	67	38	105
**R2**	72	68	140
**RG**	84	29	113
