# Supplementary material for: Differential Metabolic Rearrangements after Cold Storage Are Correlated with Chilling Injury Resistance of Peach Fruits
Source: Front Plant Sci. 2016 Sep 30;7:1478. doi: 10.3389/fpls.2016.01478 (PMC5044465; doi:10.3389/fpls.2016.01478)

Supplemental Figure 1. Visualization of metabolite-metabolite correlations of GC-MS data of each peach variety. Metabolites are grouped in modules (1 to 6) and in the same order as in Figure 2. 1: Sugars, 2: Sugar Alcohols, 3: Organic acids, 4: aminoacids, 5: Fatty acids and 6: Miscellaneous. Correlation coefficients were calculated by applying Pearson correlation. Each square represents the correlation between each metabolite heading the column and the metabolite heading the row with a color scale (color scale key at the bottom of the figure).

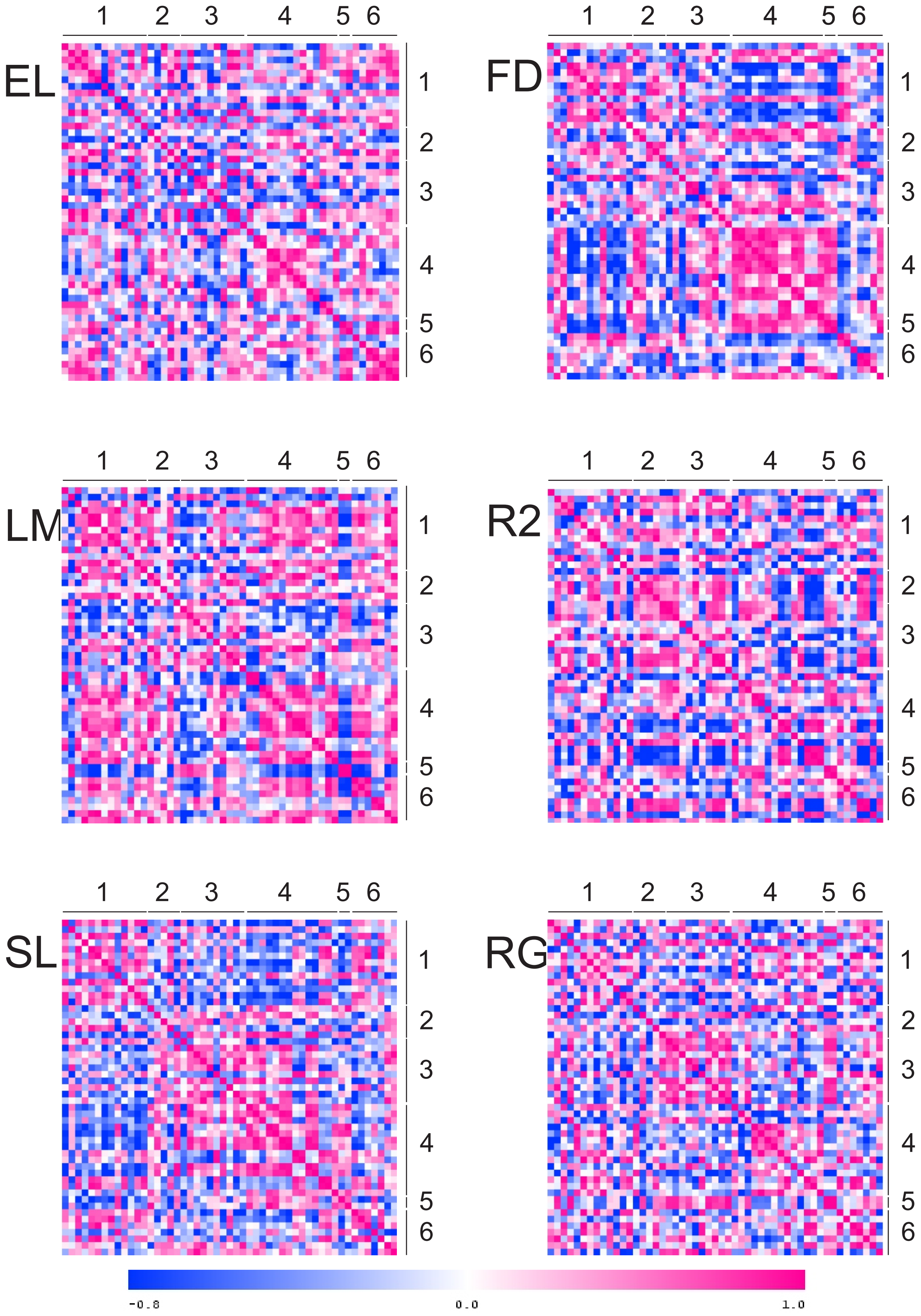

Supplement: Supplementary file 6 [file Image1.PDF]
